# Epigenetic regulation of intragenic transposable elements impacts gene transcription in *Arabidopsis* *thaliana*

**DOI:** 10.1093/nar/gkv258

**Published:** 2015-03-26

**Authors:** Tu N. Le, Yuji Miyazaki, Shohei Takuno, Hidetoshi Saze

**Affiliations:** 1 Plant Epigenetics Unit, Okinawa Institute of Science and Technology Graduate University, Onna, Okinawa 904-0495, Japan; 2 Department of Evolutionary Studies of Biosystems, School of Advanced Sciences, SOKENDAI (Graduate University for Advanced Studies), Hayama, Kanagawa 240-0193, Japan

## Abstract

Genomes of higher eukaryotes, including plants, contain numerous transposable elements (TEs), that are often silenced by epigenetic mechanisms, such as histone modifications and DNA methylation. Although TE silencing adversely affects expression of nearby genes, recent studies reveal the presence of intragenic TEs marked by repressive heterochromatic epigenetic marks within transcribed genes. However, even for the well-studied plant model *Arabidopsis* *thaliana*, the abundance of intragenic TEs, how they are epigenetically regulated, and their potential impacts on host gene expression, remain unexplored. In this study, we comprehensively analyzed genome-wide distribution and epigenetic regulation of intragenic TEs in *A*. *thaliana*. Our analysis revealed that about 3% of TEs are located within gene bodies, dominantly at intronic regions. Most of them are shorter and less methylated than intergenic TEs, but they are still targeted by RNA-directed DNA methylation-dependent and independent pathways. Surprisingly, the heterochromatic epigenetic marks at TEs are maintained within actively transcribed genes. Moreover, the heterochromatic state of intronic TEs is critical for proper transcription of associated genes. Our study provides the first insight into how intragenic TEs affect the transcriptional landscape of the *A*. *thaliana* genome, and suggests the importance of epigenetic mechanisms for regulation of TEs within transcriptional gene units.

## INTRODUCTION

Higher eukaryotic genomes harbor many transposable elements (TEs) (1–3). Due to their mobility, TEs cause various genetic changes within the host genome, from local sequence variation to large-scale genomic rearrangements, which result in great divergence in the sizes and organizations of genomes, even among closely related species (4,5). TE insertions within or close to genes also lead to the creation of novel gene regulatory elements, such as transcription start sites, splice donor/acceptor sites and polyadenylation signals, changing gene expression, and rewiring organisms transcriptional regulatory networks (5,6). In addition, exonization of TE sequences supplies the genome with a source of genetic material for evolution (7,8). Uncontrolled activity of TEs, however, causes deleterious effects, as evidenced in both plants and animals (9–13).

In host genomes, various epigenetic mechanisms, such as histone modifications and DNA methylation, have evolved to suppress activation and proliferation of TEs (14,15). Multiple epigenetic pathways are employed to deposit epigenetic silencing signals on TEs in a context-specific manner. In plants, DNA methylation in all sequence contexts can be established by the RNA-directed DNA methylation (RdDM) pathway, involving plant-specific RNA polymerase subunits NRPD1 and NRPE1, and *de novo* DNA methylase DRM. DNA methylation at CG and CHG sites is maintained by METHYLTRANSFERASE1 (MET1) and CHROMOMETHYLASE3 (CMT3), respectively (15–17). Maintenance of non-CG methylation at heterochromatic TEs also requires CHROMOMETHYLASE2 (CMT2) (18). Histone H3 lysine 9 dimethylation (H3K9me2), mediated by SET domain proteins KRYPTONITE (KYP), SUVH5 and SUVH6, facilitates binding of CMT3 to chromatin and maintains CHG methylation (15,19). The chromatin remodeler, DECREASE in DNA METHYLATION 1 (DDM1), is also required for maintenance of DNA methylation patterns at TEs in heterochromatic regions, which are distinct from targets of the RdDM pathway (18,20). In contrast, INCREASE in BONSAI METHYLATION 1 (IBM1) is responsible for keeping H3K9me2 out of genes, but not out of TEs (21).

Methylated and silenced TEs are generally excluded from genic regions, suggesting a trade-off between gene expression and TE silencing (22). However, genome-wide studies in eukaryotes have reported that there are substantial numbers of intragenic TEs in both animals and plants (23–28). Moreover, host factors INCREASE IN BONSAI METHYLATION 2 (IBM2)/ ANTI-SILENCING 1 (ASI1)/ SHOOT GROWTH 1 (SG1) and ENHANCED DOWNY MILDEW 2 (EDM2) that are specifically required for transcription of genes containing heterochormatic domain have been identified in plants (29–32). However, despite great efforts toward understanding epigenetic regulation of TEs, basic issues, such as the abundance of intragenic TEs, their epigenetic regulation and their potential impacts on expression of host genes at a genome-wide scale, have not been fully addressed, even for the well-studied plant model *Arabidopsis thaliana*.

In this work, we exploited publicly available and in-house data to address the above questions by investigating genome-wide distribution of *A. thaliana* intragenic TEs. Our analyses showed that about 3% of *A. thaliana* TEs are intragenic, mostly located within introns, that are epigenetically regulated similarly to intergenic copies. Genes harboring exonic TEs or TEs with repressive DNA methylation are often weakly expressed. Surprisingly, heterochromatic marks associated with intronic TEs are not primarily responsible for transcriptional repression of the TEs. Instead, maintenance of heterochromatic marks by epigenetic modifiers is critical for proper transcription of many host genes. Our study, therefore, provides the first insight into how epigenetic regulation of intragenic TEs contributes to genome-wide gene expression in *A. thaliana*, and suggests a significant role of epigenetic mechanisms in host resistance to TE insertion within transcriptional gene units.

## MATERIALS AND METHODS

### Genomic annotations

TE annotations were derived using two complementary approaches. First, TAIR10 release of *A. thaliana* TE annotations were retrieved from The Arabidopsis Information Resource (http://www.arabidopsis.org/). TEs from the same families located within 50 bp of each other were concatenated. TEs shorter than 50 bp were then excluded to avoid DNA fragments spuriously predicted as TEs. This resulted in a set of 19891 TEs. Second, we ran RepeatMasker (version 4.0.5; http://www.repeatmasker.org) with the Repbase library (version 20140131; http://www.girinst.org/repbase/index.html) (33). RepeatMasker-hit regions that hit simple repeats, rRNAs, satellite DNAs, centromeric repeats, low complexity regions and other composites, were excluded. We further filtered out results in which the length of the hit regions was less than 100 bp, or in which the hit regions covered less than 70% of the total length of the repeats in the library. This resulted in a set of 9517 TEs. TAIR10-based TE annotations were then compared with RepeatMasker-based annotations, resulting in a set of 7187 overlapped TEs for further analysis. On the other hand, only genes annotated as ‘protein-coding’ or ‘ncRNA’ in TAIR10 were used, which resulted in 27 600 gene annotations.

### Bisulfite sequencing data and analysis

Whole genome bisulfite sequencing (WGBS) MethylC-Seq data of various epigenetic mutant and wild-type plants were retrieved from (17). High quality reads (*q* > 28), trimmed to remove adapter effects and sequencing bias, were mapped to the *Arabidopsis* Col reference genome using Bismark (34) allowing up to two mismatches. The mapping result from wild-type sample was used to categorize TEs into high- (if CHG methylation ≥20%) or low-methylation (if CHG methylation <20%) classes. Bases covered by fewer than 3 reads were excluded, and only uniquely mapped reads were used for further analysis. Methylation levels were calculated using the ratio of $\# C/(\# C+\# T)$, as indicated in (17). Data were analyzed using MethylKit (35) and custom R scripts. Bisulfite sequencing for specific loci was performed as previously described (21).

### mRNA sequencing data and analysis

For paired-end mRNA sequencing (PE mRNA-Seq), total RNA of *ibm2* and wild-type Col were prepared as described in (29), and sequenced by the OIST Sequencing Center. Remaining mRNA-Seq data were obtained from (17,36). High quality reads were first trimmed to remove sequencing bias and adapter effects, and then mapped to the *A. thaliana* Col reference genome using Tophat (37), allowing up to 1 mismatch. Gene expression levels of the longest gene isoforms were measured using custom R scripts using only uniquely mapped reads. The downstream expression change in epigenetic mutants was calculated as described in (29) for intronic TE-containing genes if *pre-* and *post-* intronic TE read counts in wild-type, and *pre-* intronic TE read counts in corresponding mutants were ≥10. Genes showing significant defects in downstream transcription in epigenetic mutants (*P* ≤ 0.01, Fisher's exact test with Benjamini-Hochberg correction) were assigned as ‘Defect’ (or ‘D’), and the rest as ‘Non-Defect’ (or ‘ND’).

### Population genomic analysis

We used genome-wide DNA polymorphism data in 80 *A. thaliana* accessions (38) to assess the strength of selective constraints. We downloaded data from the web site of the 1001 genomes project (http://1001genomes.org). To estimate nucleotide diversity (39), we screened codons that meet following criteria: (i) no codon positions have missing data in ≥60 accessions, (ii) no accessions have premature stop codons and (iii) there are no tri- or tetra-allelic sites in any codon positions. Then, we calculated synonymous and nonsynonymous nucleotide diversity using Nei and Gojobori method (40) for each gene, excluding start and stop codons. If a gene has fewer than 100 bp synonymous change sites, it was discarded.

### Quantitative RT-PCR and 3′ RACE analysis

*cmt3-i11, ddm1-1* and *ibm2-2* were reported previously (29,41,42). *nrpe1* (SALK_029919) was obtained from the Arabidopsis Biological Stock Center. Plants were grown under long-day conditions (16h light/8h dark) at 22°C, on Murashige and Skoog (MS) agar medium in plates for two weeks. Total RNA was isolated with a Nucleo Spin RNA plant kit (TaKaRa). For cDNA synthesis, 2 μ*g* of total RNA was primed using oligo(dT) primers and reverse transcribed using a PrimeScript II 1st strand cDNA Synthesis Kit (TaKaRa). Polymerase chain reaction (PCR) amplification was performed using SYBR Premix Ex Taq II (Tli RNaseH Plus), (TaKaRa), with gene-specific primers (Supplementary Data S2). PCR reactions were carried out in a Thermal Cycler Dice Real Time System TP850 (TaKaRa). *ACTIN2* was used as an internal control. 3′ RACE for specific loci was performed as previously described (29).

## RESULTS

### Distribution of *A. thaliana* intragenic TEs

**Figure 1.**
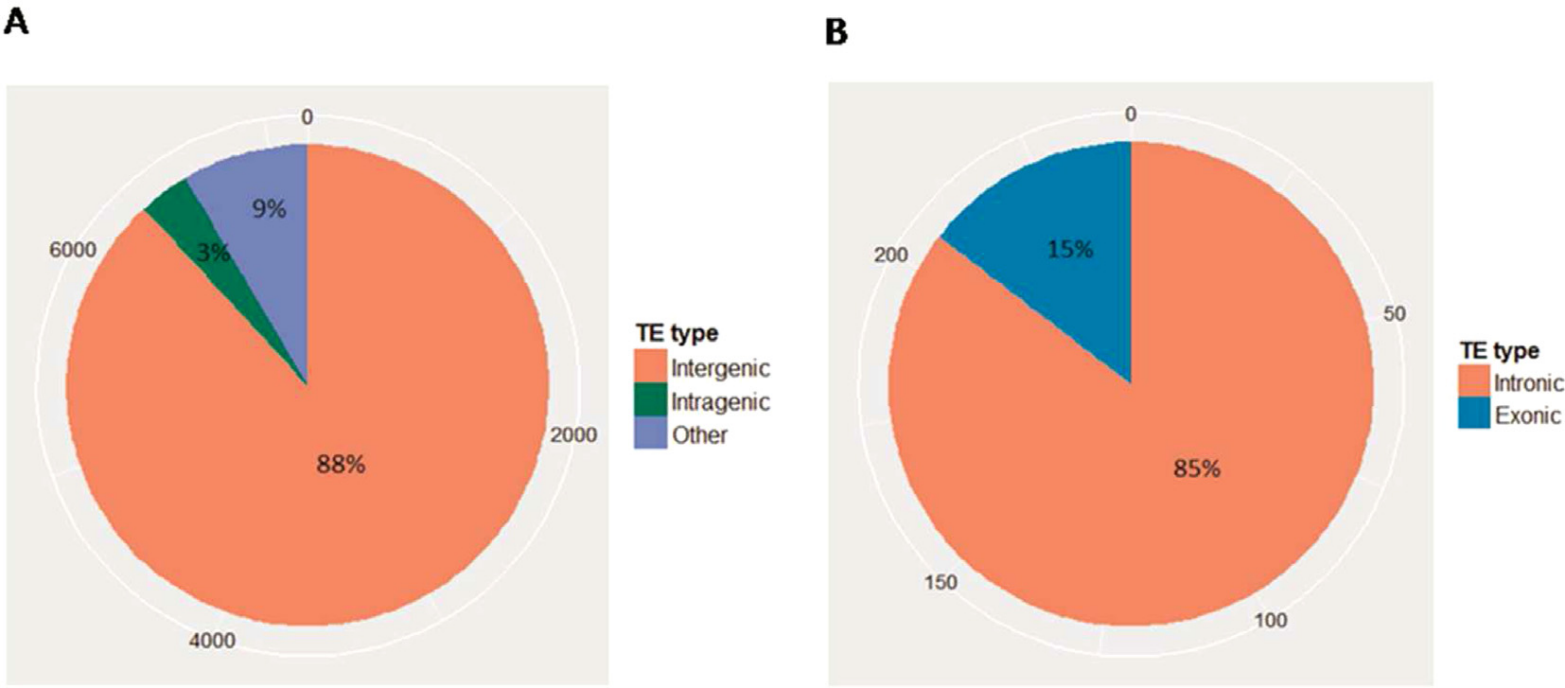
Abundance and classification of all TEs (**A**), and intragenic TEs (**B**) in the *A. thaliana* genome. The outermost circle indicates the numbers of TEs.

We have previously shown a mechanism that ensures appropriate transcription of genes with intragenic heterochromatin, which is often due to TE insertion within gene units (29). To gain a comprehensive view of intragenic TEs, we derived a set of genes and TEs from the *A. thaliana* TAIR10 genome annotation for the Columbia accession (http://www.arabidopsis.org/). Since the annotation includes many short pieces of TE sequences, TEs shorter than 50 bp were excluded to avoid the impact of poor annotation. To obtain a more rigorous TE annotation, the data set was further compared with an independently annotated, manually curated TE data set, and overlapping TEs were extracted for further analysis (for details, see Material and Methods). For gene annotation, we only adopted genes annotated as ‘protein-coding’ or ‘ncRNA’ in TAIR10. The final data set contains the annotations of 27 600 genes and 7187 TEs (including both full-length and partial fragments detected as TEs by our method), from which, 241 pairs of intragenic TEs (TEs located completely within genes) and host genes were identified. A TE that did not overlap with annotated gene was classified as an intergenic TE. Remaining TEs, that partially overlapped with or covered any gene annotation, were classified as other TEs. This procedure resulted in 6337 (88%) intergenic, 241 (3%) intragenic and 609 (9%) other TEs, respectively (Figure 1A).

Intragenic TEs were further divided into intronic and exonic TEs, according to their insertion locations in the gene body. A TE was considered intronic if more than 95% of its length was within an annotated intron, or exonic if not. This classification resulted in 206 (85%) intronic and 35 (15%) exonic TEs, in 214 host genes (Figure 1B, Supplementary Data S1). Of these genes, 182 (85%), 28 (13.1%) and 4 (1.9%) harbored only intronic TEs, only exonic TEs, or both, respectively (Supplementary Figure S1). The data indicate that introns are more tolerant to TE insertions than exons (*P* < 1*e* − 15, goodness-of-fit *χ*^2^-test), and that intronic and exonic insertions are almost exclusive.

**Figure 2.**
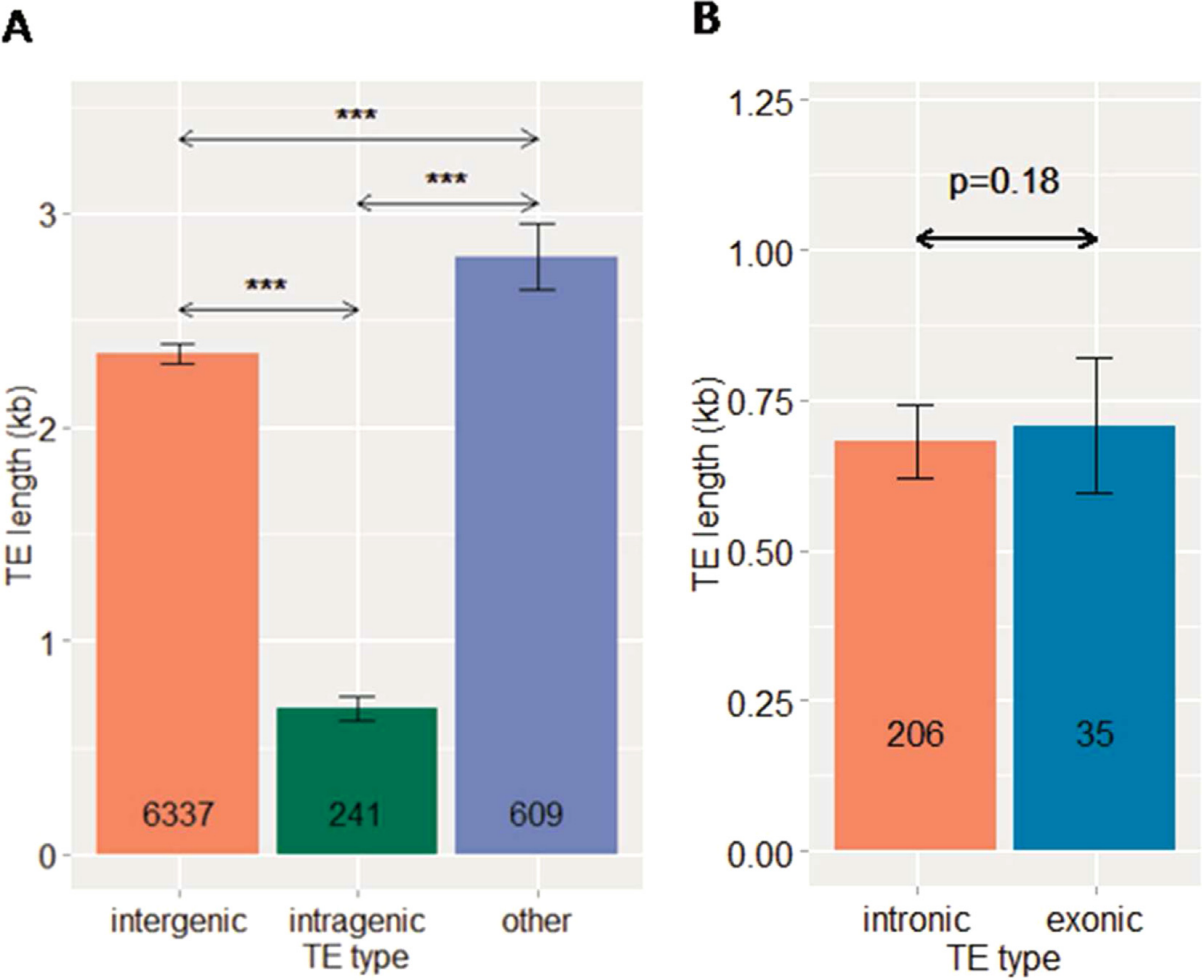
Size difference between intergenic, intragenic, and other TEs (**A**), and between intronic and exonic TEs (**B**). *P* values were given by the *Mann-Whitney U (MWU)* test. (***) corresponds to *P* < 0.0005. Numbers inside bar plots indicate the total numbers of TEs in each category. Error bars represent mean ± SE.

We found that intragenic TE sequences were significantly shorter than intergenic TEs (Figure 2A). About 17% were equal or longer than 1 kb, which was about three times less than intergenic TEs (51%) (Supplementary Figure S2A). The results suggest that, after integration, full-length TEs have degenerated and been preferentially purged from gene bodies by selection force, likely due to their negative effects on host gene expression (22,43). On the other hand, there was no significant difference in the lengths of intronic and exonic TEs (Figure 2B). Surprisingly, other TEs were longer than both intergenic and intragenic TEs (Figure 2A), suggesting that these loci could be long intergenic TEs parts of which were misannotated as overlapped genes.

As TEs belong to distinct families that differ in structure, transposition and silencing mechanisms (2,18), we then asked if there is any family preference for TE insertions within genes. Among the most abundant families, *Gypsy* TEs were biased against (1.7%), and *Mariner* were preferentially inserted (5.4%) within genes compared with intergenic regions (17% and 1.5%, respectively) (*P* < 0.001, 2 × 2 contingency *χ*^2^-test, Bonferroni-correction) (Supplementary Figure S2B).

### Epigenetic silencing of intragenic TEs and gene expression

TEs in plants are silenced by multiple epigenetic pathways (15). Active genes are often methylated specifically at CG sites, whereas silenced TEs are methylated in all contexts including non-CG methylation, a hallmark of inactive heterochromatin (44,45). Genes located close to silenced TEs tend to have low expression (22); thus we further investigated DNA methylation at intragenic TEs and its potential impacts on the expression of associated genes.

**Figure 3.**
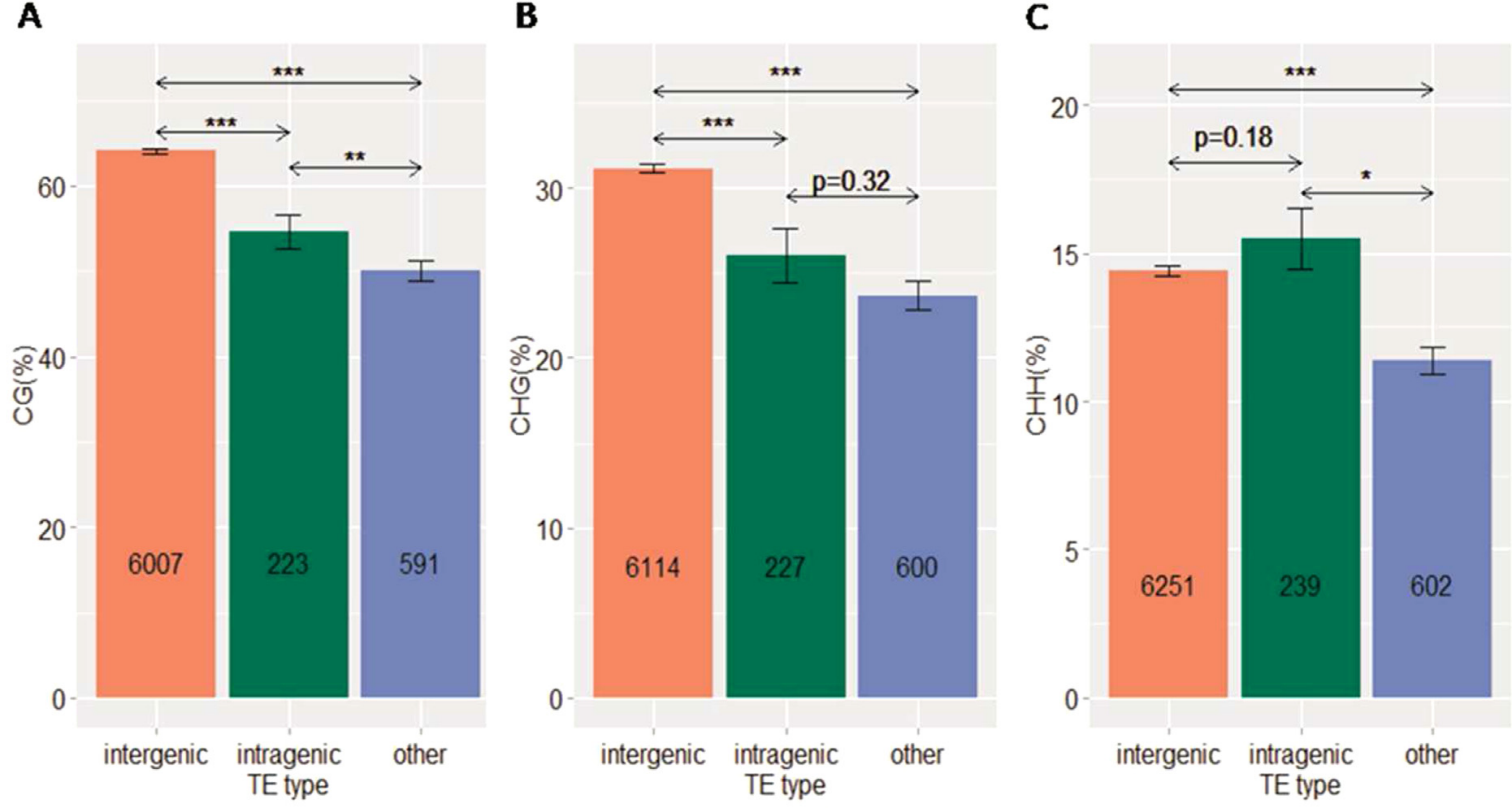
Difference of DNA methylation between intergenic, intragenic and other TEs in CG (**A**), CHG (**B**) and CHH (**C**) contexts. *P* values were given by the *MWU* test. (*), (**), (***) correspond to *P* < 0.05, 0.005 and 0.0005, respectively. Numbers inside bar plots indicate the total numbers of TEs in each category. TEs that lacked methylation were excluded. Error bars represent mean ± SE.

**Figure 4.**
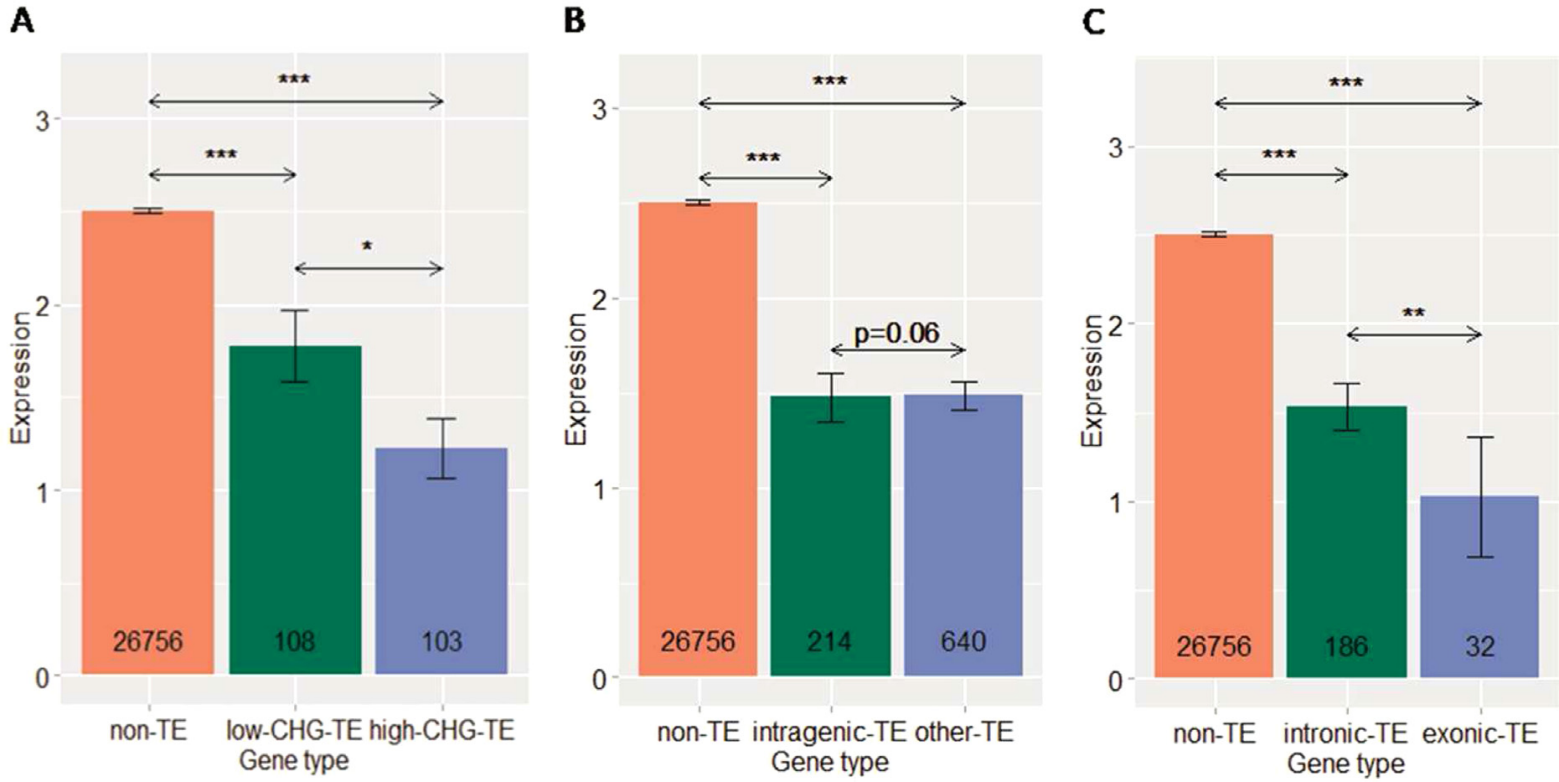
Negative impact of intragenic TEs and their methylation on gene expression (*log*_2_(*RPKM* + 1)) in *A. thaliana*. (**A**) Expression of genes containing high-CHG-methylated TEs, low-CHG-methylated TEs and genes without TE insertions. (**B**) Expression of genes containing intragenic TEs, overlapped with other TEs and genes without TE insertions. (**C**) Expression of genes harboring exonic TEs and intronic TEs, and genes without TE insertions. (*), (**), (***) correspond to *P* < 0.05, 0.005 and 0.0005, the *MWU* test, respectively.

Our DNA methylation analysis showed that intragenic TEs were less methylated than intergenic TEs in CG and CHG contexts (Figure 3), while this difference was not observed between intronic and exonic TEs (Supplementary Figure S3). Interestingly, other TEs, despite being longer than both intergenic and intragenic TEs, were much less methylated, in almost all sequence contexts (Figure 3). These results suggest that there is strong selection against methylated TEs in intragenic and proximal regions, which could be explained by the negative effect of DNA methylation on gene expression. This is indeed supported by our data, in which genes containing more highly methylated TEs are expressed at significantly lower levels than genes containing less methylated TEs, and these genes, in turn, are expressed at significantly lower levels than genes without TE insertions (Figure 4A). Other TEs, as expected, had a negative impact on the expression of nearby genes, comparable to that of intragenic TEs (Figure 4B).

Possibly, the lower expression level of genes with TE insertions could be explained by the process of pseudogenization (46,47). To test this hypothesis, we calculated the ratio of nonsynonymous and synonymous nucleotide diversity as an indicator of selective constraints. We utilized genome-wide DNA polymorphism data in *A. thaliana* (38) and found that, on average, genes with and without intragenic TEs did not differ significantly (0.416 versus 0.439; *P* > 0.8, permutation test). Thus, there is no evidence of a relaxation of selective constraints on genes bearing intragenic TEs.

We further predicted that insertions within coding regions (e.g. exons) should have a stronger impact than insertions within non-coding regions (e.g. introns). Indeed, genes bearing exonic TEs were expressed at significantly lower levels than genes bearing intronic TEs, and both were expressed significantly less than genes without TE insertions (Figure 4C).

### Regulation of DNA methylation at intragenic TEs

**Figure 5.**
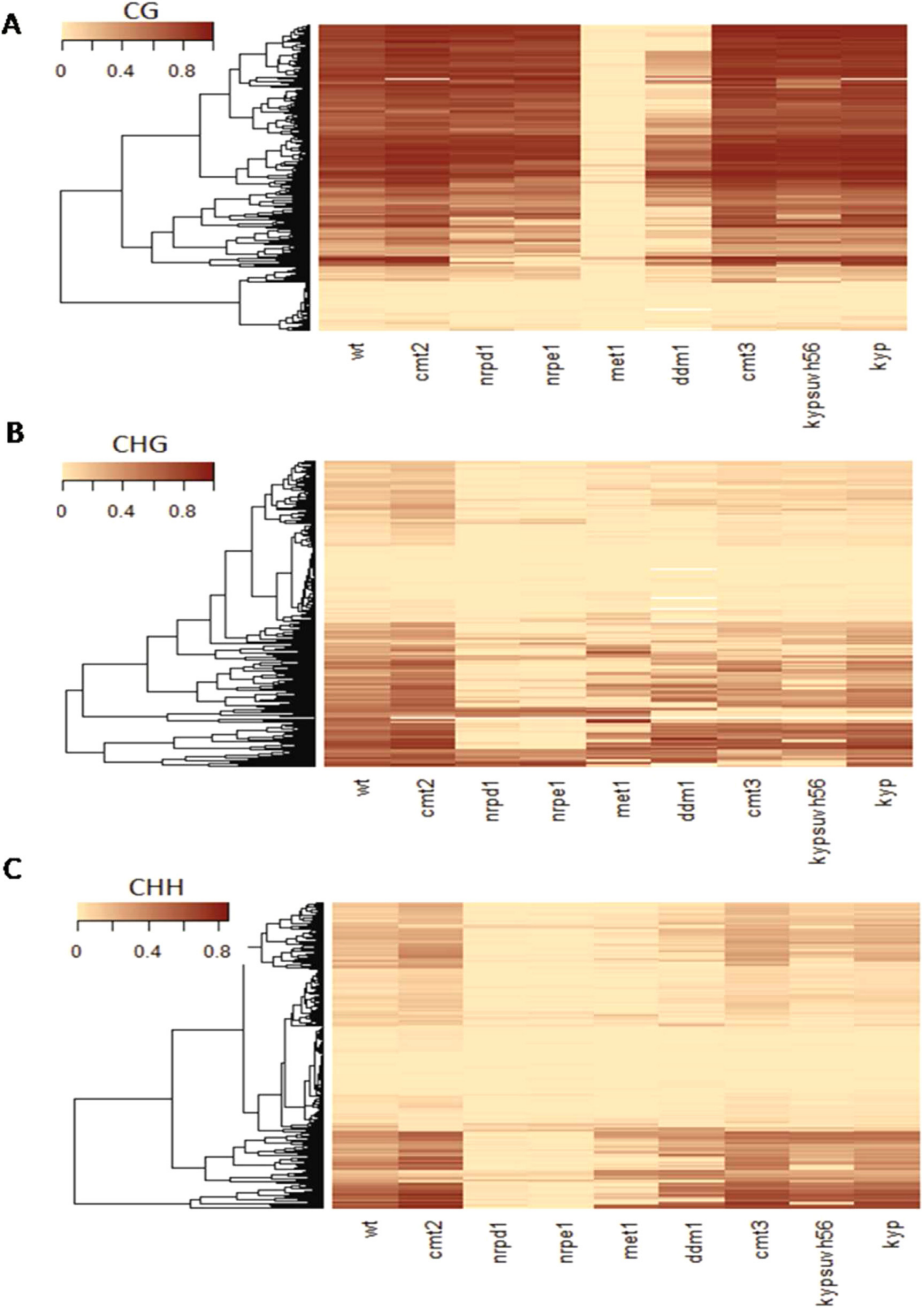
Heatmap of CG (**A**), CHG (**B**) and CHH (**C**) methylation at *A. thaliana* intragenic TEs in epigenetic mutants. Rows and columns represent intragenic TEs and indicated genotypes, respectively. Rows were organized by hierarchical clustering on methylation levels of TEs in wild-type plant. TEs that lacked methylation were excluded.

Given that intragenic TEs are targeted by CG and non-CG methylation, we further analyzed epigenetic factors required for regulation of intragenic TEs using publically available data (17). CG methylation of intragenic TEs was severely reduced in mutants of methyltransferase MET1 and chromatin remodeler DDM1 (Figure 5A, Supplementary Figure S4). Consistent with a previous report (17), CHG methylation in intragenic TEs was reduced in mutants of genes regulating genome-wide CHG methylation, e.g. *cmt3, kyp*, and in the triple mutant of H3K9 methylases *kypsuvh56* (Figure 5B, Supplementary Figure S5). Mutant of CMT2, which regulates methylation at CHG and CHH contexts in a DDM1-dependent manner (18,20), did not have a strong effect on non-CG methylation of intragenic TEs (Figure 5B and C). On the other hand, mutants of RdDM pathway components, such as *nrpd1* and *nrpe1*, strongly reduced non-CG methylation, consistent with a previous report that non-CG methylation of short TEs located in euchromatic regions is predominantly regulated by RdDM (18). However, non-CG methylation in a subset of intragenic TEs was also regulated by *cmt3, kypsuvh56*, or *ddm1*, the factors required for methylation in long TEs enriched with H3K9me2 (Figure 5B and C, Supplementary Figure S6). These results demonstrate that DNA methylation in intragenic TEs is differentially regulated by both RdDM-dependent and -independent mechanisms, as observed in short euchromatic TEs and long heterochromatic TEs in the *Arabidopsis* genome (18). In addition, we observed that DNA methylation of intragenic TEs was not affected by mutation in the *IBM1*, which ectopically induces non-CG methylation at actively transcribed gene bodies, but not at TEs (48,49) (Supplementary Figures S7 and S8). Also, intragenic TE sequences were clearly distinguished by methylation peaks from surrounding genic regions. These data suggest that, similarly to intergenic TEs, intragenic TEs are specifically recognized by the epigenetic modifiers that maintain CG and non-CG methylation, even though they are located within the actively transcribed regions.

### Intronic heterochromatic marks affect gene transcription

Loss of DNA methylation in epigenetic mutants results in transcriptional activation of intergenic TEs. Surprisingly, intronic TEs were not strongly activated in epigenetic mutants, including *met1* and *ddm1* (Supplementary Figure S9). This is likely due to truncation/degeneration of TE promoters, but also suggests additional roles of intragenic heterochromatic marks beyond transcriptional silencing of TEs. Given the observation that genes containing intronic TEs were relatively highly expressed (Figure 4B), we hypothesized that heterochromatic epigenetic marks carried by intronic TEs might be important for proper transcription of associated genes. Recent studies have provided evidences supporting this hypothesis, suggesting that epigenetic factors are important for proper transcription of exons downstream of heterochromatic domains (29,32). A similar effect was also observed at specific loci in mutants defective in genome-wide DNA methylation (50,51).

**Figure 6.**
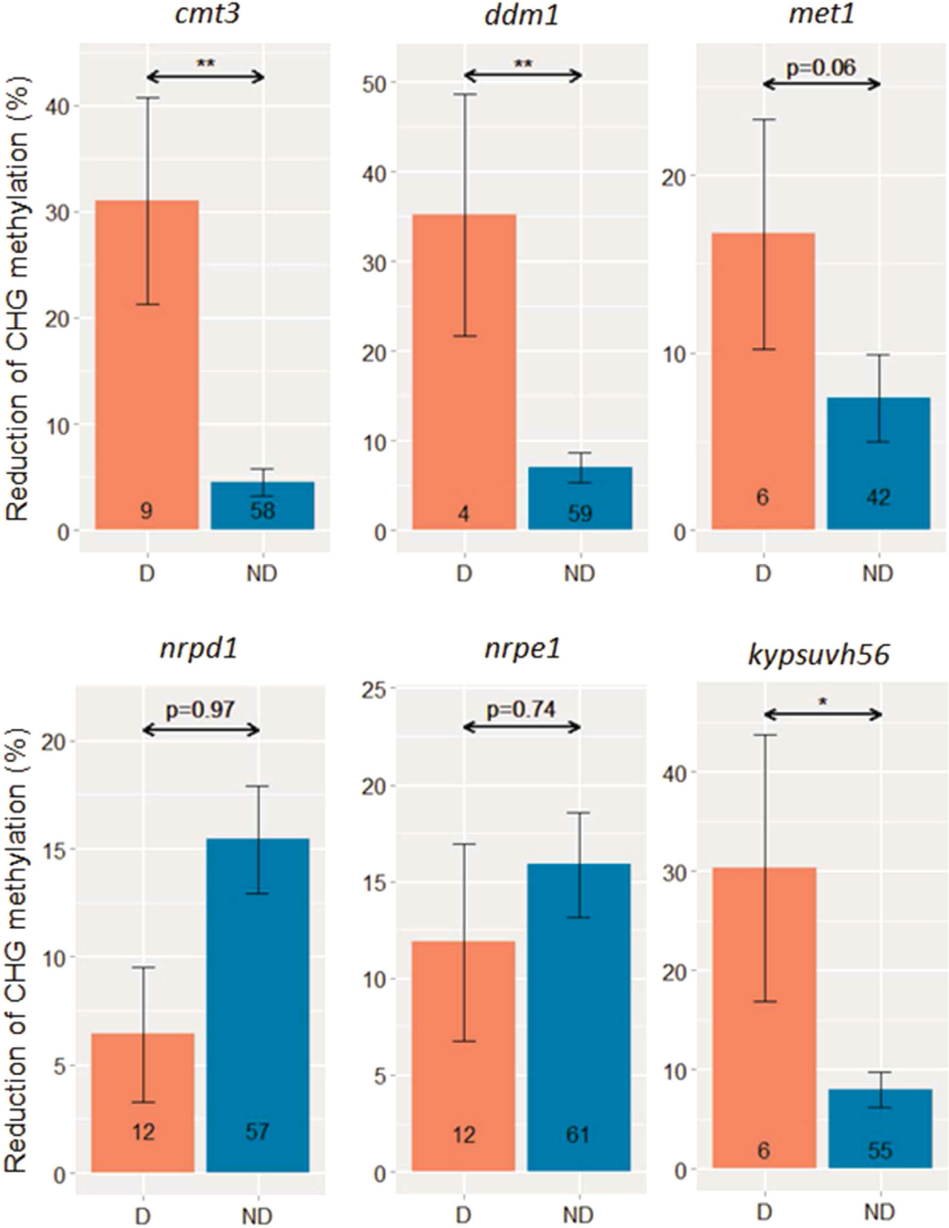
Reduction of CHG methylation of intronic TEs in epigenetic mutants is associated with transcription defects (D, defect; ND, No-Defect). Multiple TEs in a single gene were analyzed independently. TEs that lacked methylation were excluded. Genes with fewer than 10 reads mapped to *pre*- and *post*-intronic TE regions in wild-type, or in *pre*-intronic TE regions in mutants, were also excluded. Numbers inside bar plots indicate the total numbers of intronic TEs in each category. *P* values were given by the *MWU* test. (*) and (**) correspond to *P* < 0.05 and 0.005, respectively. Error bars represent mean ± SE.

**Figure 7.**
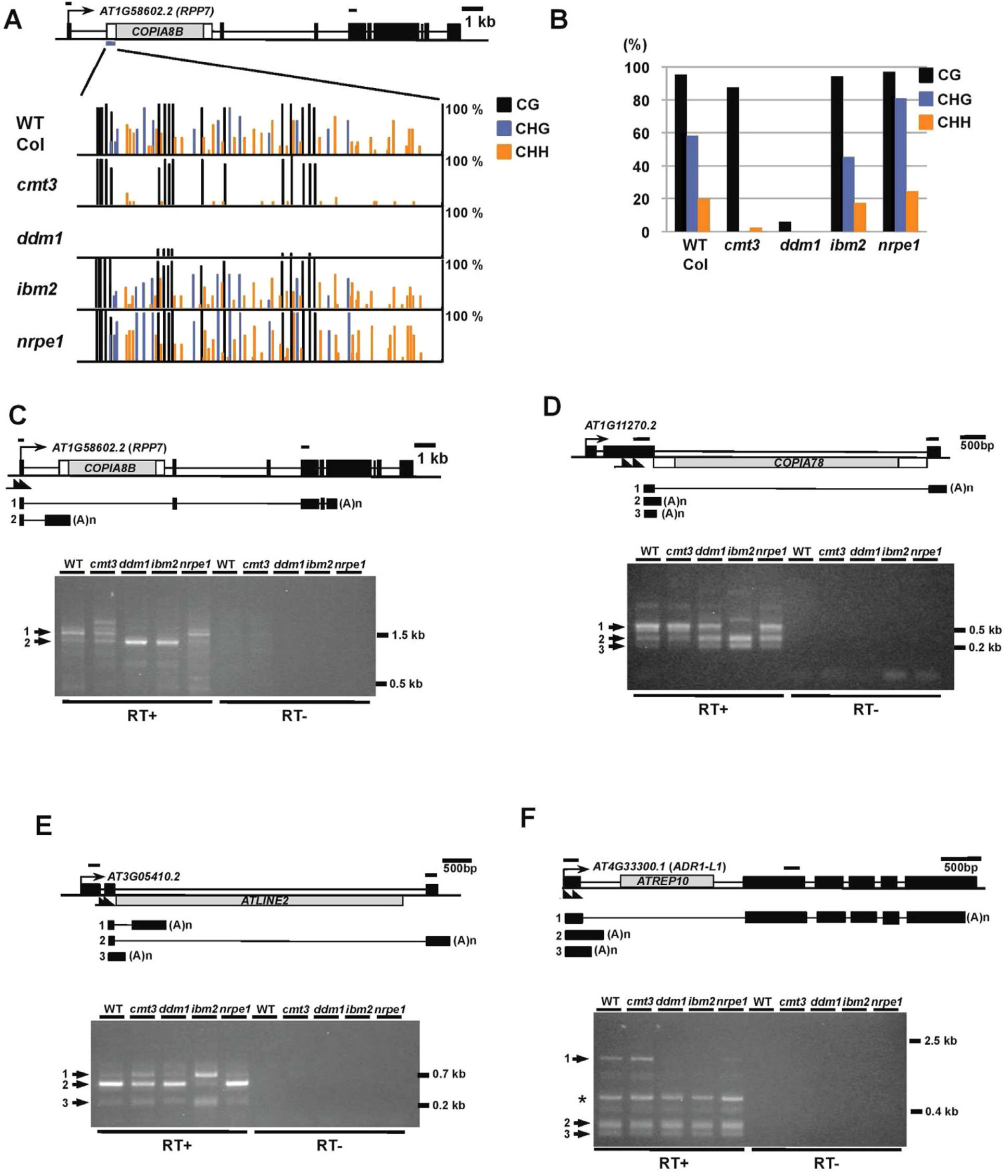
Epigenetic factors are required for proper expression of genes containing intronic TEs. (**A**) Bisulfite sequencing analysis for DNA methylation at the *RPP7* (*AT1G58602*) locus in epigenetic mutants. Exons and TEs are indicated by black and gray boxes, respectively. Twelve independent clones were sequenced for the indicated genotypes. (**B**) Summary of bisulfite analysis in (A). (C–F) 3′ RACE of genes containing intronic TEs. (**C**) Upper panel; structure of the *RPP7* locus and polyadenylated mRNA variants detected by 3′ RACE. Exons and spliced introns confirmed by sequencing analysis are shown as black boxes and lines, respectively. Note that a full-length transcript was not recovered, and only prematurely terminated transcripts (transcript 1 or 2) were identified by 3′ RACE, likely because the predicted full-length cDNA is relatively long (4.3 kb). Primers used for 3′ RACE are indicated by arrows, and those for qPCR in Supplementary Figure S13 are indicated as horizontal bars. Lower panel; gel picture of DNA fragments amplified by 3′ RACE. DNA fragments indicated by arrowheads were cloned and sequenced, and representative clones were shown in the upper panel. (**D**) 3′ RACE of the TE enclosing gene *AT1G11270* shown as (C). (**E**) 3′ RACE of the TE enclosing gene *AT3G05410* shown as (C). (**F**) 3′ RACE of the TE enclosing gene *AT4G33300* (*ADR1-L1*) shown as (C). Asterisk represents unidentified fragments.

We thus investigated genome-wide relationships between CHG methylation, a hallmark of heterochromatin controlled by H3K9me (19), of intronic TEs and transcription of associated genes in different epigenetic mutants using public data (17,36). Except in mutants of the RdDM pathway, e.g. *nrpd1* and *nrpe1*, reduction of CHG methylation of intronic TEs in *cmt3, ddm1, met1* and *kypsuvh56* were highly correlated with transcriptional defects of exons downstream of the intronic TEs (Figure 6). A similar result was also obtained when possible bias caused by multiple TE insertions within a single gene was removed by keeping only one representative TE for each gene (Supplementary Figure S10). Moreover, intronic TEs within genes showing transcription defects tended to be longer and more highly methylated than intronic TEs within genes with no transcription defects (Supplementary Figures S11 and S12), suggesting that heterochromatic epigenetic modifications, including CHG methylation and H3K9me, are especially important for transcription of genes containing long heterochromatic TEs. To validate the RNA-Seq analysis, we further performed experimental analysis, by selecting genes harboring either highly or slightly methylated intronic TEs that showed transcription defects in at least one of the following mutants: *cmt3, ddm1, ibm2* and *nrpe1* (Supplementary Data S2). Quantitative PCR analysis confirmed that genes harboring highly methylated intronic TEs tend to show transcription defects in expression downstream of TE sequences (Supplementary Figure S13), that include *RPP7* (*AT1G58602*) or *ADR1-L1* (*AT4G33300*), genes encoding nucleotide-binding leucine-rich repeat (NB-LRR) proteins involved in plant immune responses (51,52) (Figure 7A). It is also worth noting that *RPP7, AT3G05410* and *AT1G11270* contain relatively long TEs (about 5 kb), and *ATLINE2* in *AT3G05410* and *COPIA78* (also known as *ONSEN*) in *AT1G11270* maintain intact open reading frame (ORF) and full-length TE sequences (Figure 7). Reduction of CHG methylation in retrotransposon sequences in *RPP7* and *AT3G05410* loci was associated with transcription defects downstream of the TE sequences (Figure 7B, C, D and E, Supplementary Figure S14). On the other hand, the *ADR1-L1* locus showed a complex response, as the reduction of downstream transcription of the *ADR1-L1* was not associated with changes in DNA methylation in the TE (Figure 7F, Supplementary Figure S14) in *ddm1*, which suggests additional epigenetic regulation by DDM1. *ibm2* did not affect DNA methylation in these loci, consistent with a hypothesis that IBM2 acts downstream of heterochromatic epigenetic modifications (29). 3′ RACE experiments using polyA-tailed mRNA demonstrated that transcripts over TE sequences were reduced in four of the selected loci, and instead, shorter transcripts prematurely polyadenylated around the 5′ region of TE sequences were increased in the mutants (Figure 7C, D, E and F). The results suggest that heterochromatic epigenetic modifications in intronic TEs promote splicing of intron encompassing TE sequences, and/or prevent premature polyadenylation. Taken together, our data suggest that maintenance of heterochromatic state at intronic TEs by epigenetic factors is essential for proper transcription of genes containing TEs.

## DISCUSSION

Intragenic TEs are commonly found in higher eukaryotes, especially those with large genomes (3,25). Although the contribution of intragenic TEs on structural changes of host genes and their influence on host gene function have been extensively studied, epigenetic regulation of intragenic TEs and its impacts on gene activities remain to be elucidated. This study provides the first comprehensive examination of the genome-wide epigenetic landscape of intragenic TEs and its direct relationship to host gene transcription in *A. thaliana*, one of the best model organisms for epigenetic studies. We revealed that intragenic TEs are generally short, and preferentially inserted within intronic regions. Although intronic and exonic TEs are epigenetically regulated similarly to each other, and to intergenic TEs, they impact gene expression differently. In particular, CHG methylation of intronic TEs is highly associated with proper transcription of host genes, suggesting a critical role of intragenic heterochromatin in host tolerance to deleterious TE insertions within transcriptional gene units.

### Genome tolerance to intragenic TEs

TEs within gene units disrupt gene structure, and epigenetic silencing of TEs negatively affects expression of nearby genes (4,22). Because of these detrimental effects, TEs are generally purged from gene-rich regions during evolution (22). Still, many of them have been able to escape elimination and have spread into gene bodies (Figure 1A), indicating that those intragenic TEs have lost or acquired some properties that allow them to remain within transcriptional gene units.

First, a majority of intragenic TEs are truncated or degenerated relics of ancestral sequences (≥80% of intragenic TEs are shorter than 1 kb, Supplementary Figure S2A), and therefore likely do not maintain cryptic regulatory signals, such as splice donor/acceptor sites and polyA signals, that would affect mRNA structure and transcriptional regulation of associated genes. It has been suggested that unequal homologous recombination and illegitimate recombination mechanisms are responsible for removal of TE sequences from the genome, which are most active in euchromatic regions (53,54). These mechanisms, in combination with negative selection against long, intact TEs, would affect the structure and distribution pattern of intragenic TEs.

Secondly, intragenic TEs are often less methylated than intergenic TEs (Figure 3), consistent with a previous report that DNA methylation is a major constraint on TEs close to genes (22). Also, most of the insertions occur within introns (Figure 1B), which tend to be excluded from mature mRNAs after splicing, and likely do not affect host gene functions (29). Those structural changes and insertional selection would allow TEs to remain within gene units under selective pressure.

Third, epigenetic mechanisms can mask the effects of a TE sequence within a gene body. Previous studies showed that host epigenetic mechanisms involving IBM2 and EDM2 allow enhanced splicing of heterochromatic introns formed by TE insertions (29,32). In this study, we showed that maintenance of heterochromatic epigenetic modifications, including CHG methylation and H3K9 methylation, in intronic TEs is essential for masking the TEs, which requires CMT3 and DDM1 (Figure 6, Supplementary Figures S10 and S11). Intriguingly, intronic heterochromatin seems to have properties distinct from those of intergenic heterochromatin, as IBM2 is preferentially recruited to intronic, but not to intergenic heterochromatin (29). Recent studies for plants with large genomes revealed that genes containing intronic TEs are common, which might contribute to the expansion of genome size (27,28,55). Interestingly, expression levels of those genes with intronic TEs are often comparable to those without TEs, suggesting the presence of epigenetic masking mechanisms as observed in *A. thaliana*. Thus, host epigenetic mechanisms might be able to specifically neutralize deleterious effects of intronic TEs, resulting in increased tolerance to heterochromatic TEs within intronic regions.

### RdDM-dependent and independent DNA methylation in intragenic TEs

The RdDM pathway controls non-CG methylation at short euchromatic TEs and edges of heterochromatic TEs (18). Our study showed that non-CG methylation of intronic TEs is regulated by both RdDM-dependent and -independent pathways, with a dominant role for RdDM in facilitating CHH methylation (Figure 5). However, RdDM-dependent CHG methylation of intronic TEs was not strongly correlated with host gene transcription, unlike other epigenetic mutants such as *cmt3* and *ddm1* (Figure 6, Supplementary Figures S10 and S11). This suggests that RdDM in genic regions may have roles other than transcriptional repression of TEs or promoting transcription over TEs. It has been shown that targeting of TEs by unique siRNAs is linked to TE sequence deletion (30), suggesting that RdDM may contribute to selective removal of TEs from genic regions.

### Functional impact of intragenic TEs

TEs located close to genes often acquire regulatory functions that are controlled by their epigenetic states. In particular, epigenetic changes of TEs located in promoter regions directly affect downstream gene expression (56–58). In contrast, functional impacts of epigenetic regulation of intragenic TEs within gene bodies are less clear. Our study demonstrated that maintenance of the heterochromatic state of intronic TEs is important for proper host gene expression at dozens of loci (Figure 6, Supplementary Figures S10, S11 and S13). In mice, CG methylation of intronic TEs at several imprinted gene loci regulates utilization of alternative polyA signals, resulting in production of different transcript isoforms from paternal and maternal alleles (59,60). Similarly, in *Arabidopsis*, full-length transcription of *IBM1* is controlled by DNA methylation of one of its introns, the region likely to be conserved before speciation of *A. thaliana* (29,50). This and previous studies showed that maintenance of heterochromatin at intronic TEs is important for proper expression of immune response genes *RPP7* and *ADR1-L1* (Figure 7) (51). On the other hand, a recent study showed that non-CG methylation at intronic TEs induced by vernalization is associated with up-regulation of a flowering gene *VRN1* in winter wheat (61). These results suggest that epigenetic states of intragenic TEs might modulate expression of genes responsive to environmental signals and biotic stresses. Although understanding of the functional relevance of intronic TEs requires further analyses, these data suggest a potential role of intragenic TEs in the process of alternative splicing and gene activation via epigenetic regulation.

## CONCLUSION

We have demonstrated that even in the TE-poor *A. thaliana* genome, many TEs are present within intragenic regions. The heterochromatic state is maintained in intragenic TEs by epigenetic modifiers that mask deleterious effects of TE insertion within the gene body. Whether intragenic TEs within the set of genes become adaptive, however, remains unclear. Analyses of plants with larger genomes could provide further insights into the functional relevance and contribution of intragenic TEs to genome evolution.

## ACCESSION NUMBERS

Sequencing data have been deposited in the DDBJ Sequence Read Archive under accession codes: DRA002305 (Col mRNA-seq) and DRA002306 (*ibm2* mRNA-seq).

## SUPPLEMENTARY DATA

Supplementary Data are available at NAR Online.

SUPPLEMENTARY DATA
