# Supplementary material for: Epigenetic regulation of intragenic transposable elements impacts gene transcription in Arabidopsis thaliana
Source: Nucleic Acids Res. 2015 Mar 26;43(8):3911–21. doi: 10.1093/nar/gkv258 (PMC4417168; doi:10.1093/nar/gkv258)
Supplement: SUPPLEMENTARY DATA [file supp_43_8_3911__index.html]

Epigenetic regulation of intragenic transposable elements impacts gene transcription in Arabidopsis thaliana — Epigenetic regulation of intragenic transposable elements impacts gene transcription in Arabidopsis thaliana — SUPPLEMENTARY DATA 

# Epigenetic regulation of intragenic transposable elements impacts gene transcription in *Arabidopsis* *thaliana*

## SUPPLEMENTARY DATA

**Files in this Data Supplement:**

- SUPPLEMENTARY DATA
- SUPPLEMENTARY DATA
- SUPPLEMENTARY DATA
