# Supplementary material for: Epigenetic regulation of intragenic transposable elements impacts gene transcription in Arabidopsis thaliana
Source: Nucleic Acids Res. 2015 Mar 26;43(8):3911–21. doi: 10.1093/nar/gkv258 (PMC4417168; doi:10.1093/nar/gkv258)

Supplementary Figure S1. Abundance of genes harboring TE insertions in *A. thaliana*. Red is genes with insertions in exons. Blue is genes with insertions within introns. Green is genes with insertions in both exons and introns. The outermost circle presents the numbers of genes.

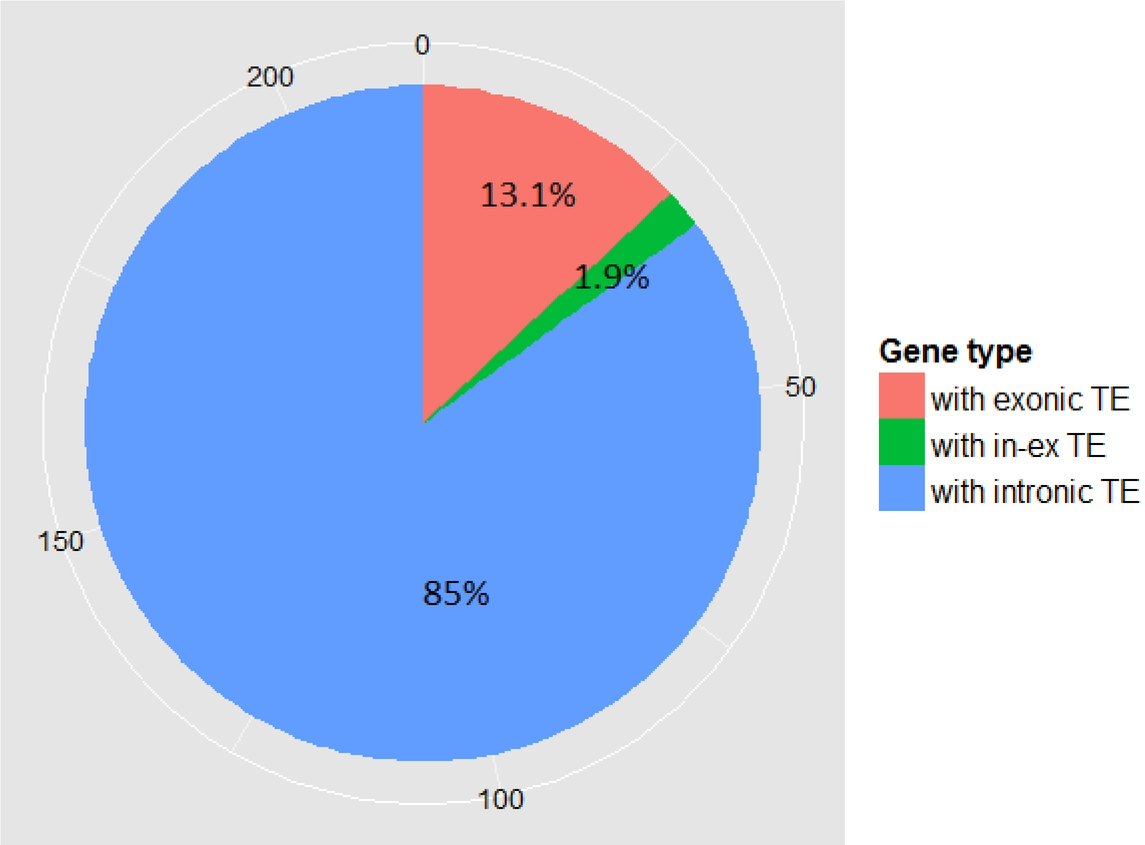

Supplementary Figure S2. Distribution of intergenic and intragenic TEs in *A. thaliana* categorized by TE length (A), and TE family (B).

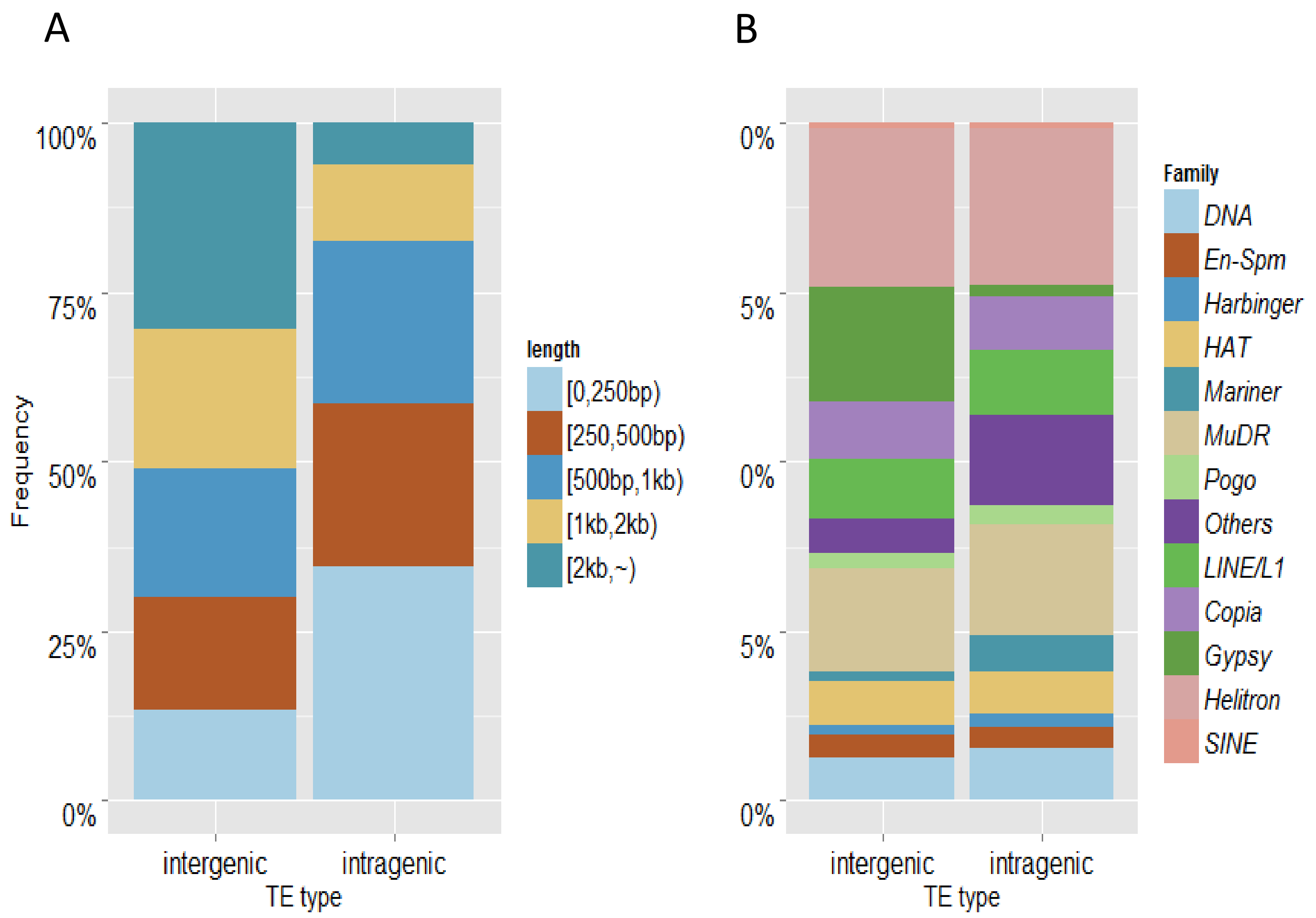

Supplementary Figure S3. Difference of DNA methylation between intronic and exonic TEs in CG (A), CHG (B), and CHH (C) contexts. *p* values were given by the *MWU* test. Numbers inside bar plots correspond to the total numbers of TEs in each category. TEs that lacked methylation were excluded. Error bars represent *mean±se*.

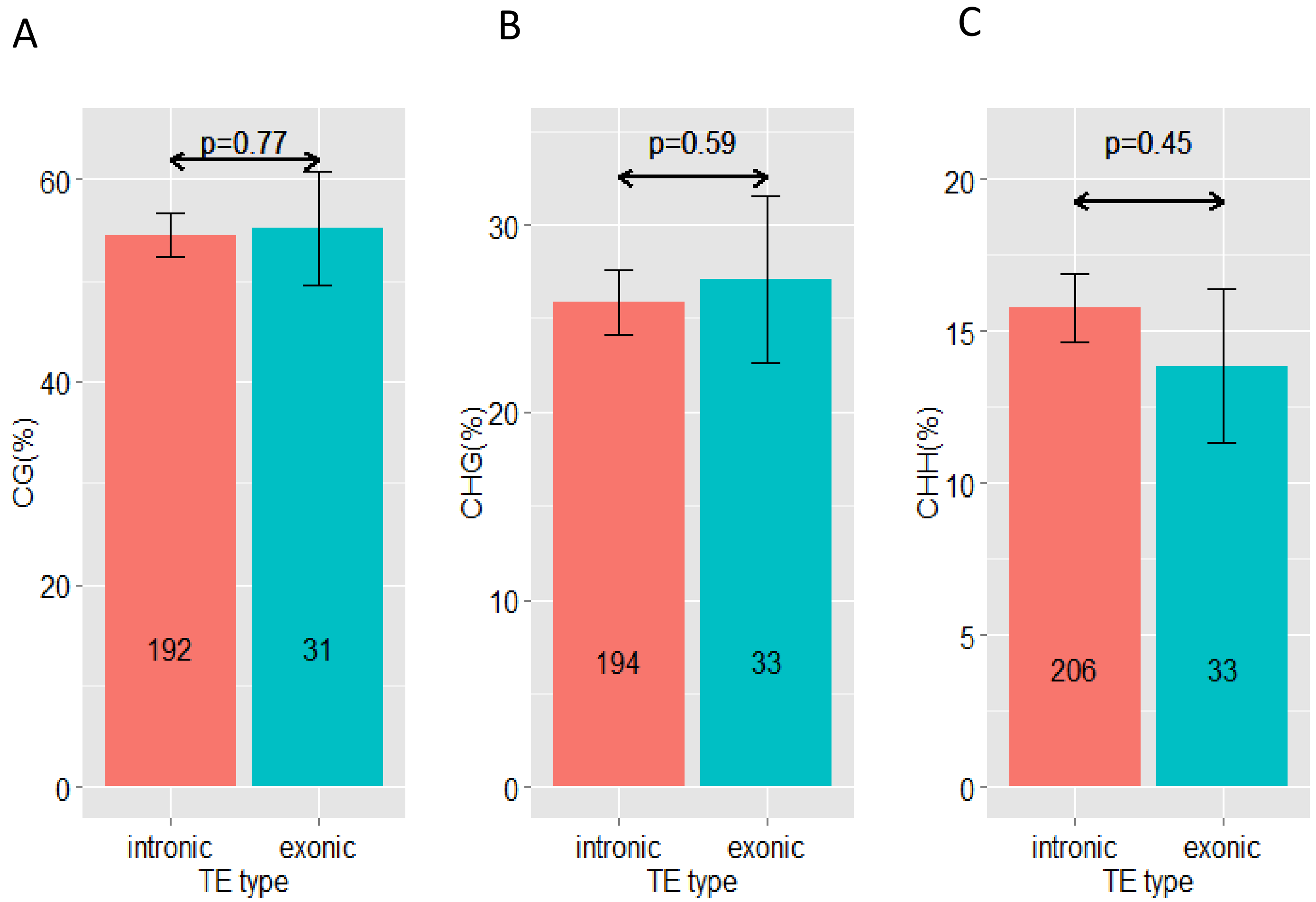

Supplementary Figure S4. Average distribution of DNA methylation over TEs in wild-type Col (wt; black lines), *ddm1* (red lines), and *met1* (blue lines). Methylation profiles at TEs and surrounding regions of the same lengths were measured by first dividing these regions into 10 equal-length bins, and then smoothed using *locally weighted scatterplot smoothing (LOWESS)* method.

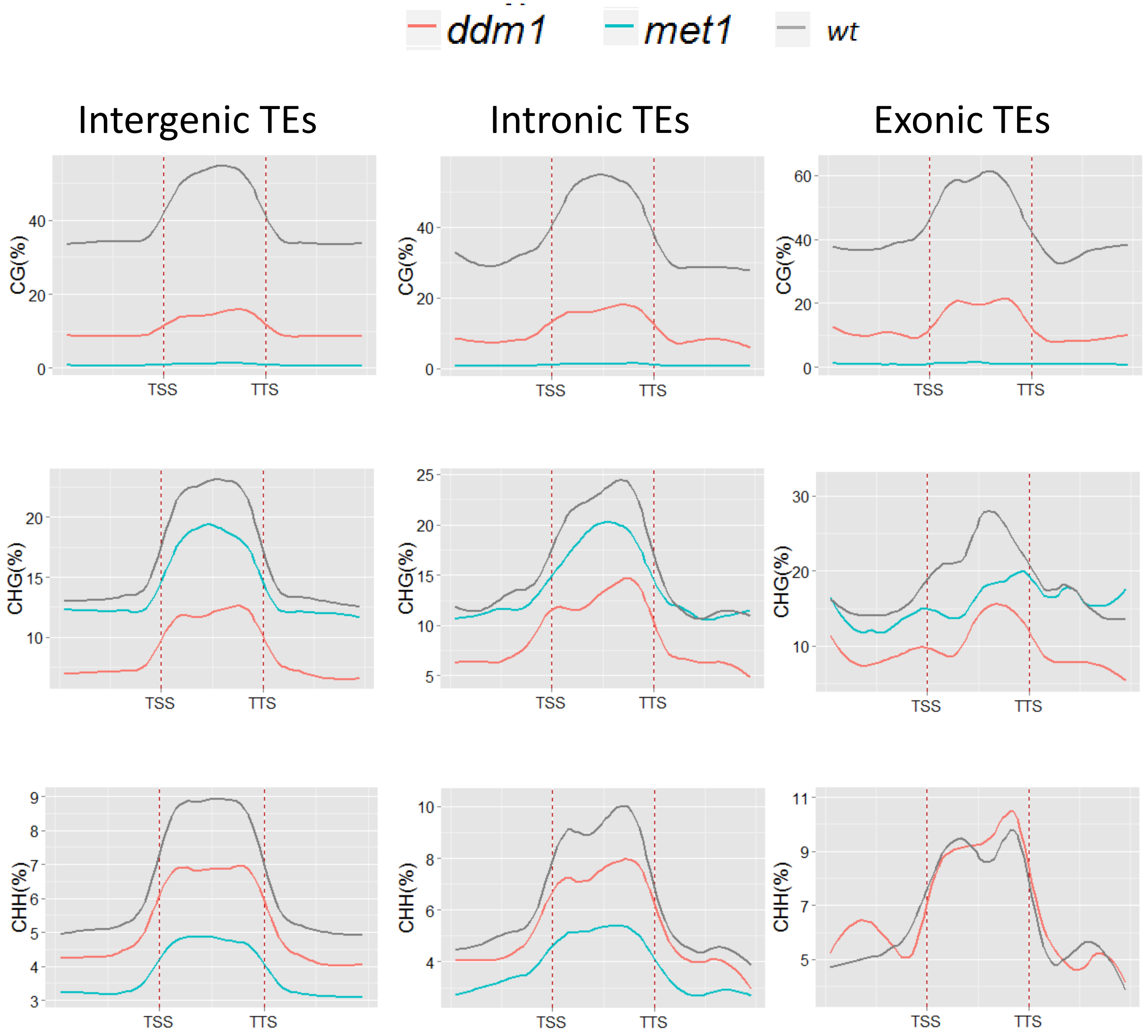

Supplementary Figure S5. Average distribution of DNA methylation over TEs in wild-type Col (wt; black lines), *cmt3* (red lines), *kyp* (green lines), and *kypsuvh56* (blue lines) as described in Supplementary Figure S4.

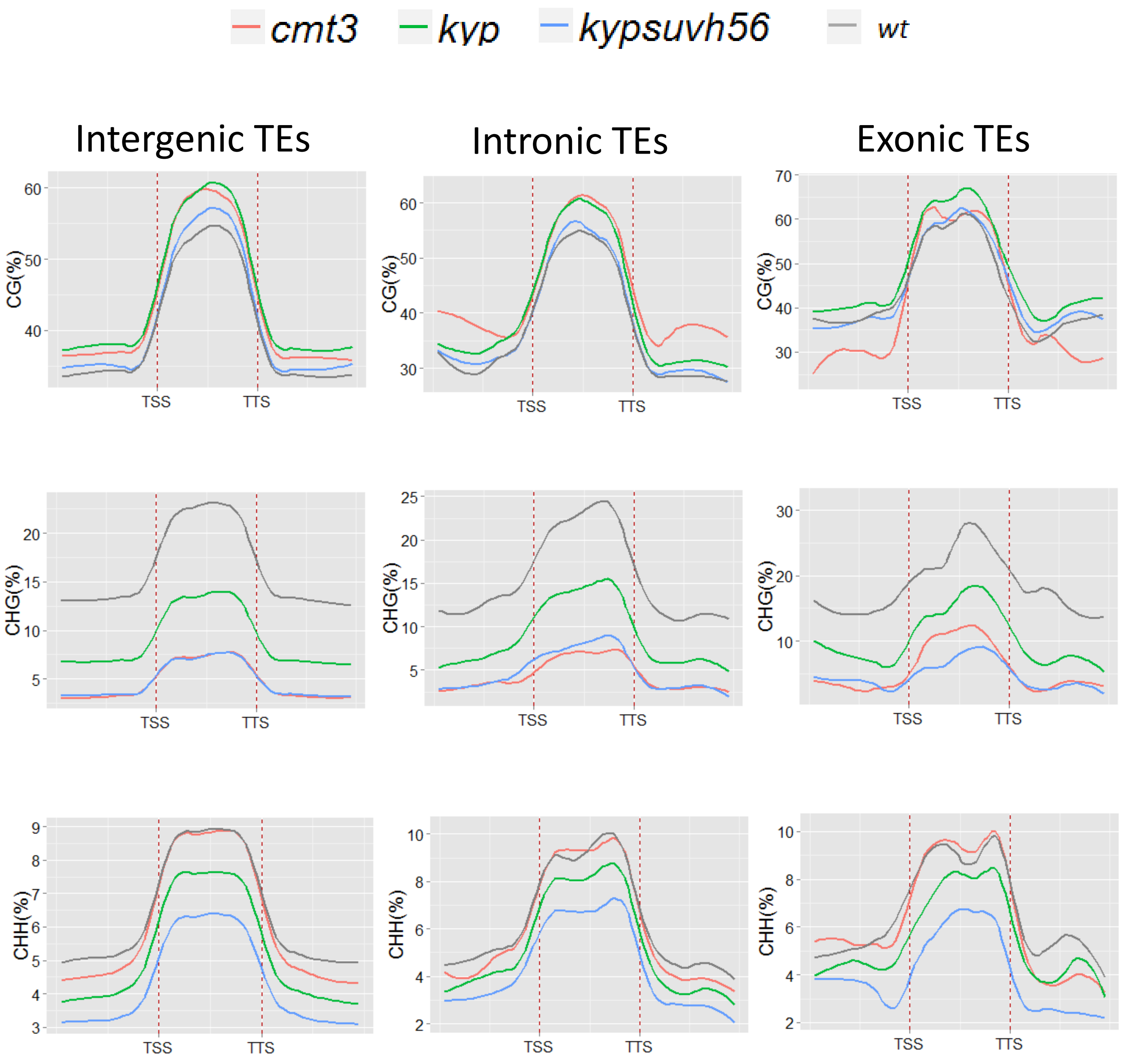

Supplementary Figure S6. Average distribution of DNA methylation over TEs in wild-type Col (wt; black lines), *nrpd1* (red lines), and *nrpe1* (blue lines) as described in Supplementary Figure S4.

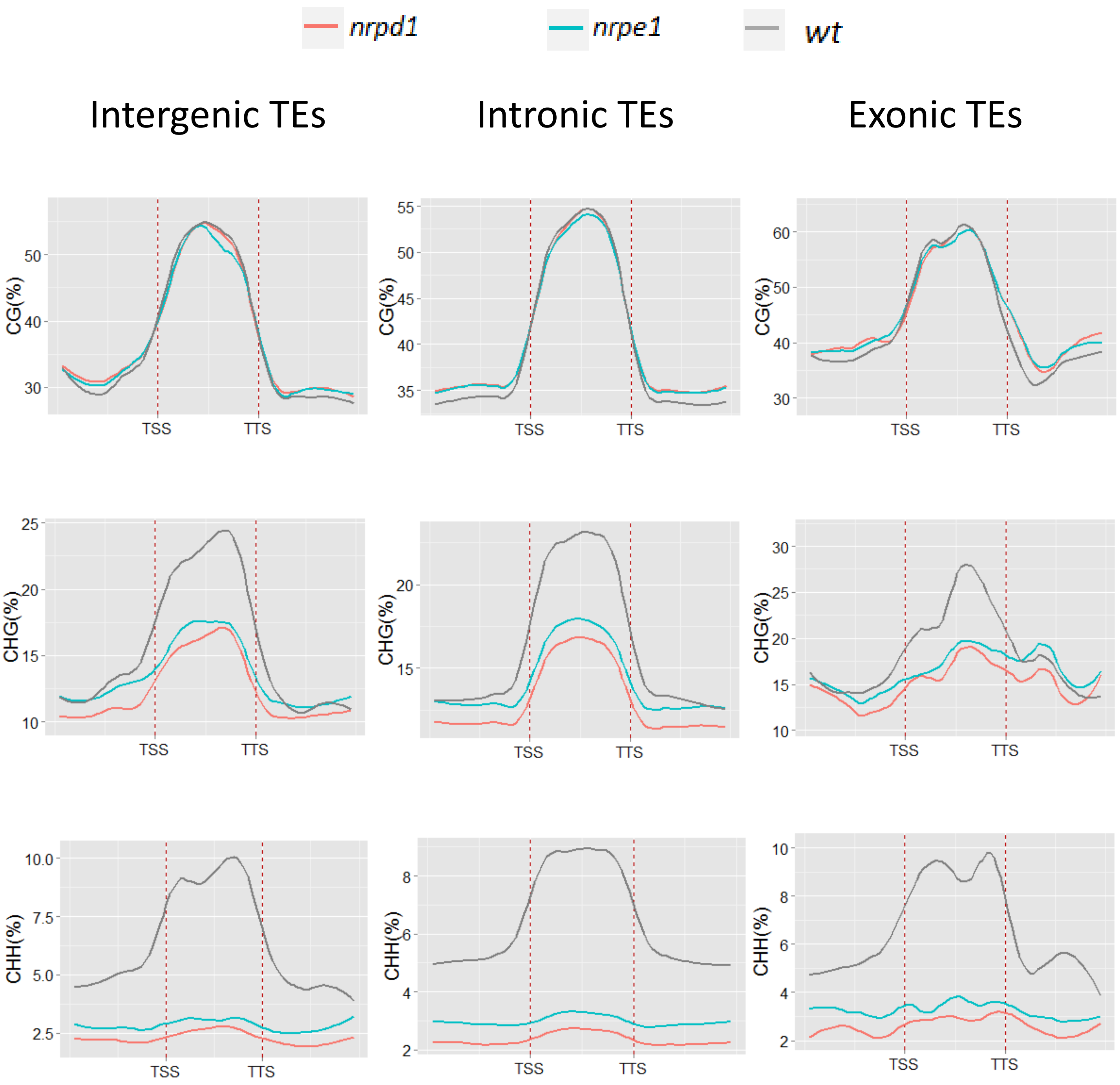

Supplementary Figure S7. Change of CHG methylation at exonic and intronic TEs in *ibm1*. Methylation (%) of TEs in wild-type Col (wt; x-axis) and *ibm1* (y-axis) are shown.

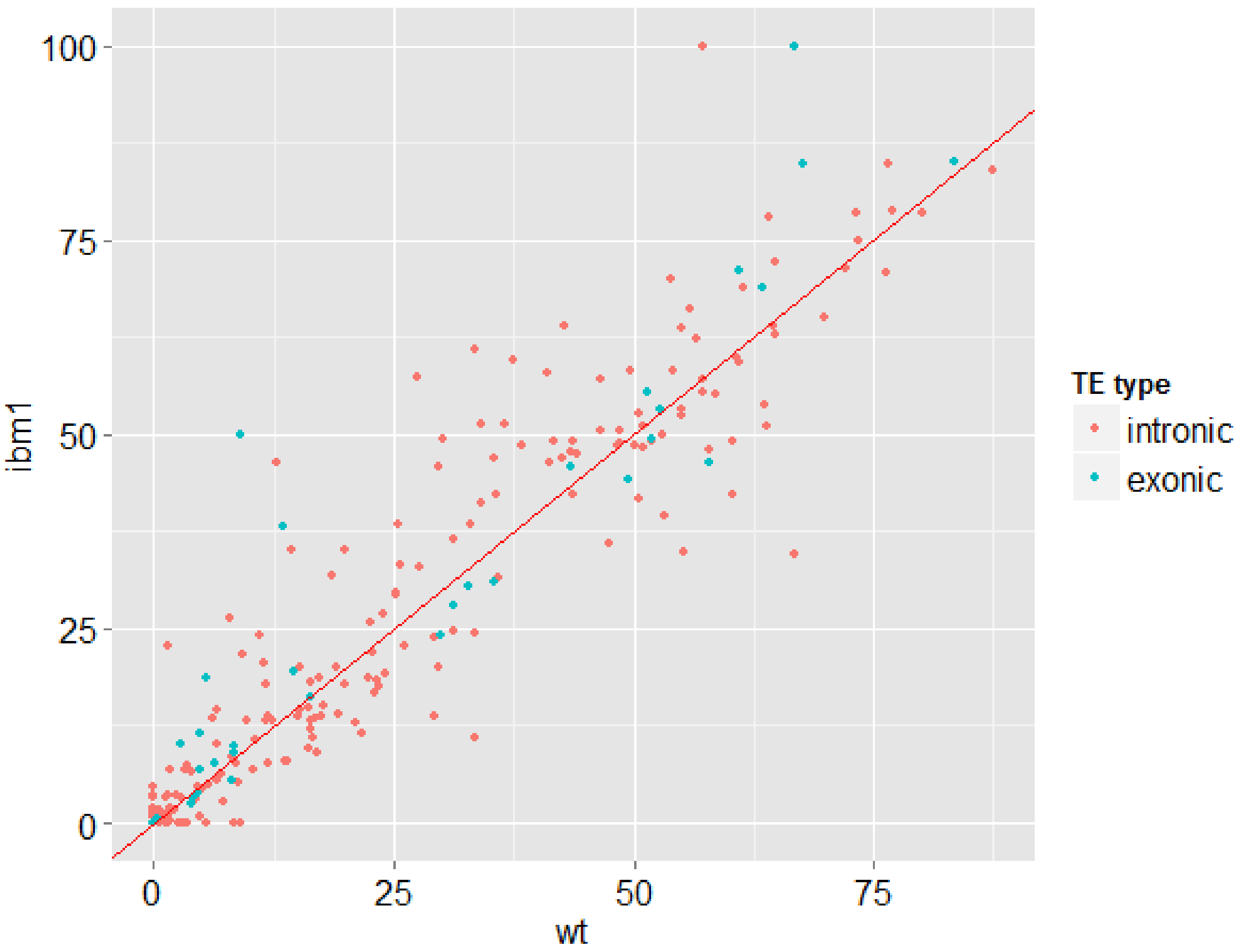

Supplementary Figure S8. Average distribution of DNA methylation over TEs in wild-type Col (wt; black lines) and *ibm1* (red lines) as described in Supplementary Figure S4.

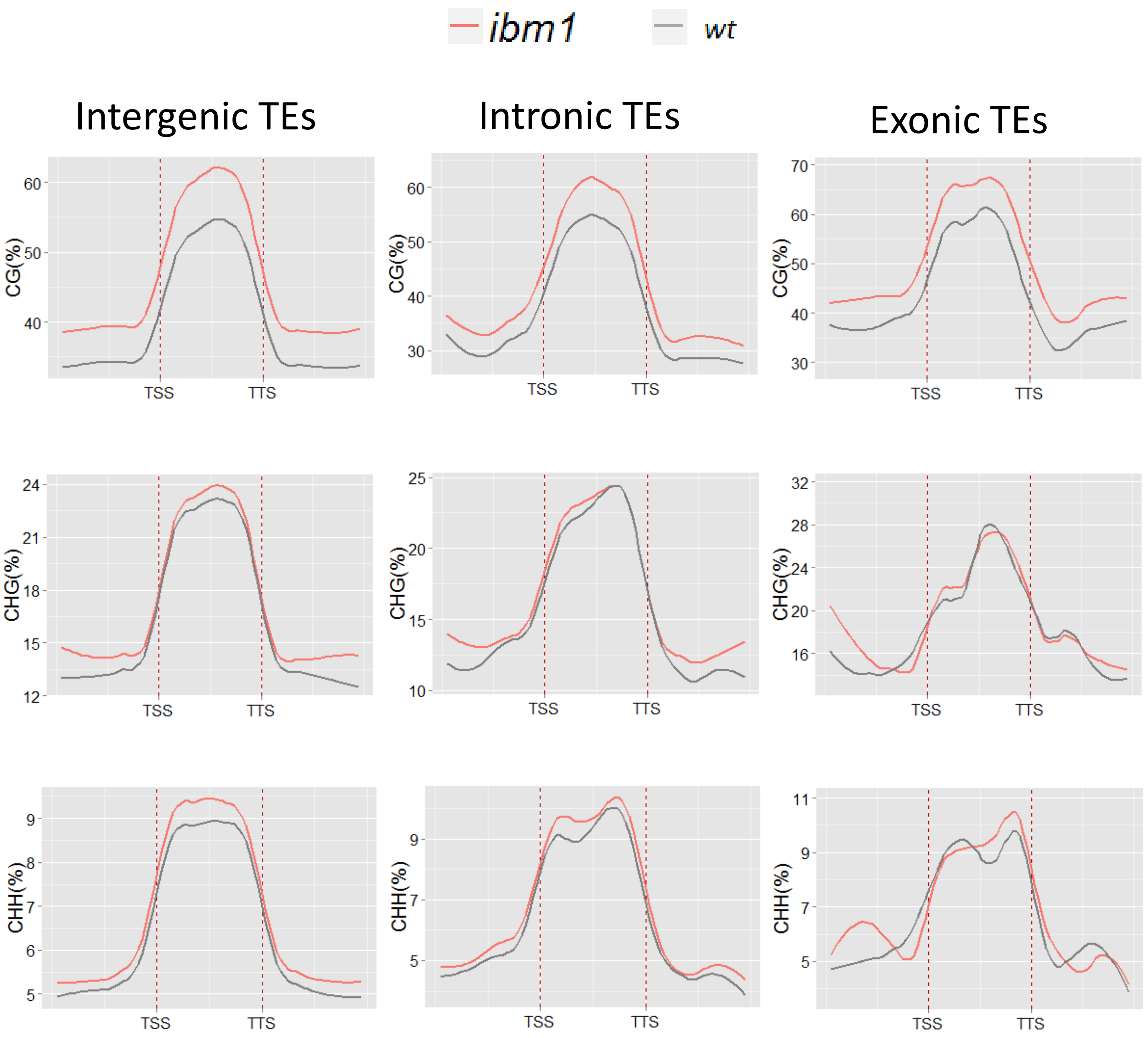

Supplementary Figure S9. Change in expression of intergenic and intronic TEs by epigenetic mutants. Expression values (exp) were normalized to RPKM scale and log2(exp+1) was presented for wild-type Col (wt; x-axis) and epigenetic mutants indicated in the panels (y-axis).

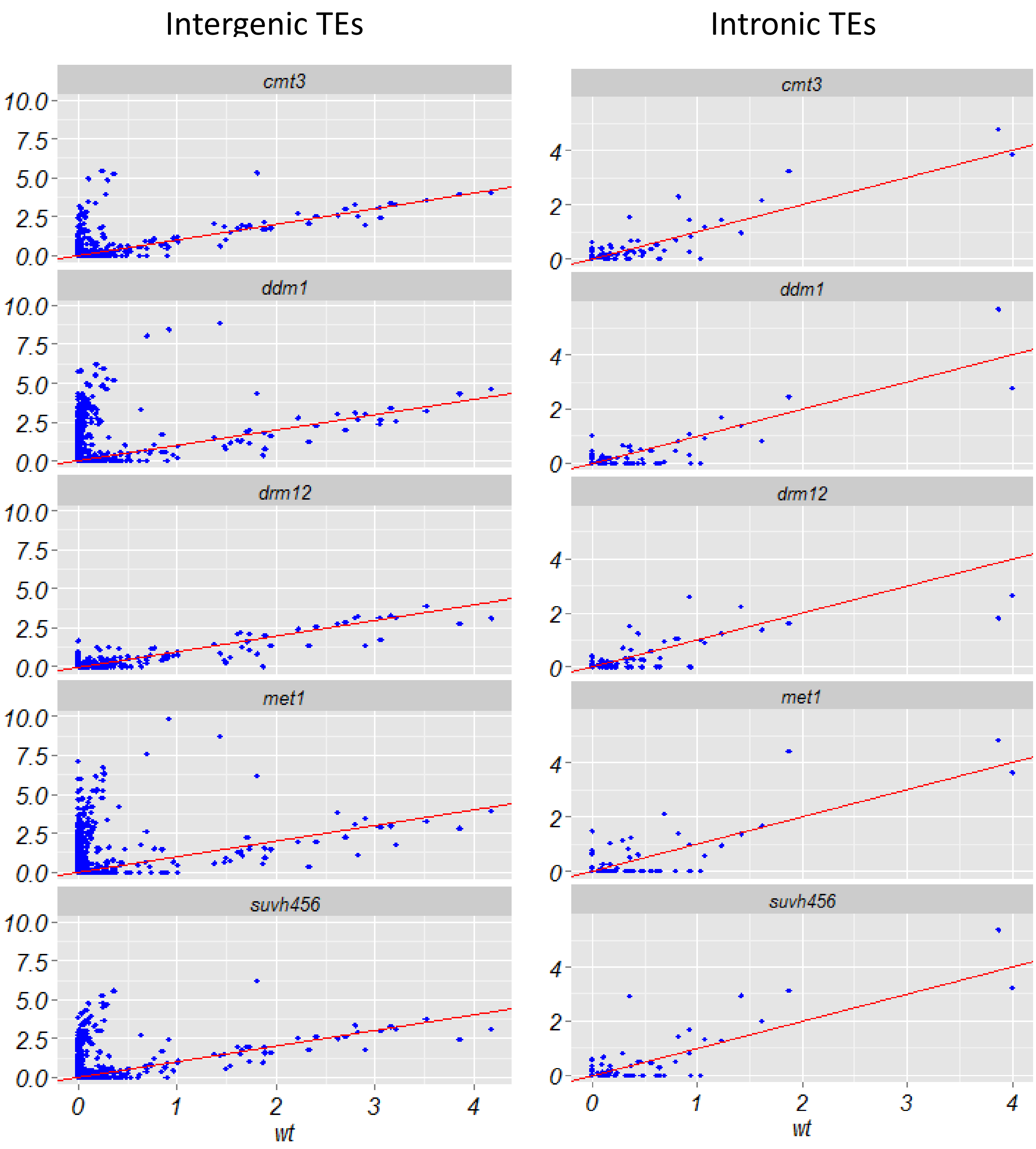

Supplementary Figure S10. Reduction of CHG methylation of intronic TEs in epigenetic mutants is associated with transcription defect (D, defect; ND, No-Defect). The analysis is similar to Figure 6, except only the longest intronic TE in a gene was adopted.

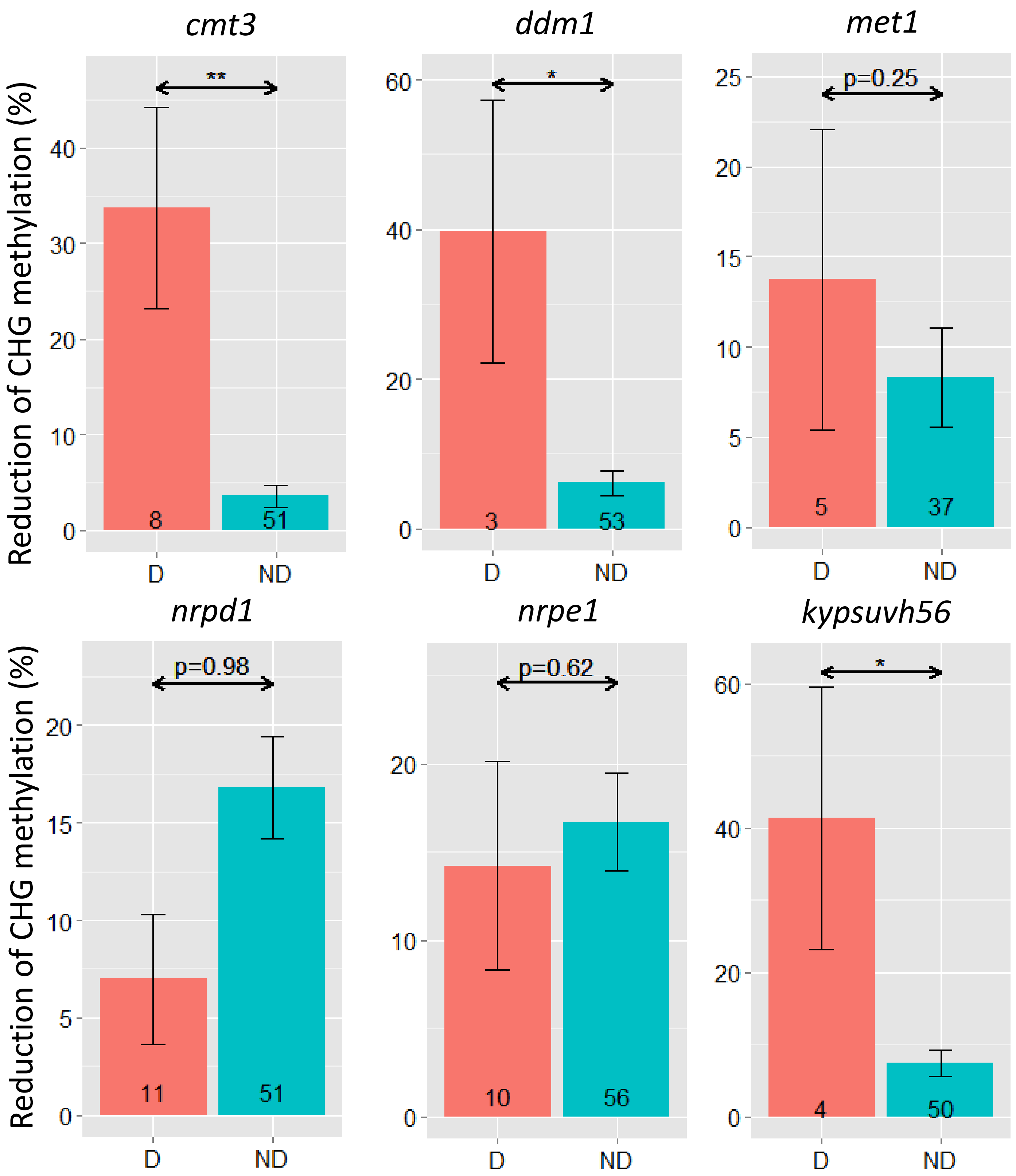

Supplementary Figure S11. Genes containing TEs with high CHG methylation show transcription defect in epigenetic mutants. D, defect; ND, No-Defect.

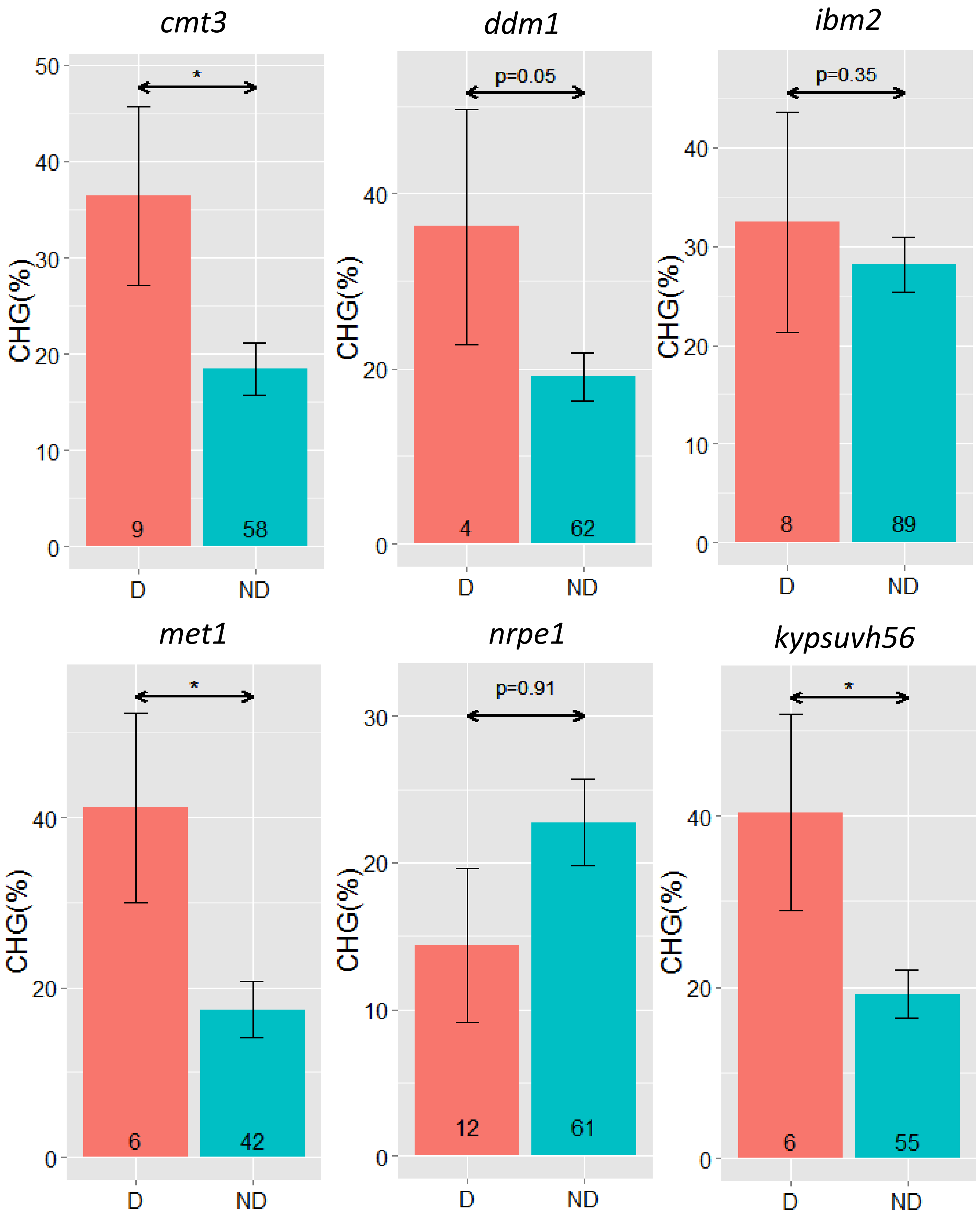

Supplementary Figure S12. Genes containing long TEs show transcription defect in epigenetic mutants. D, defect; ND, no-defect.

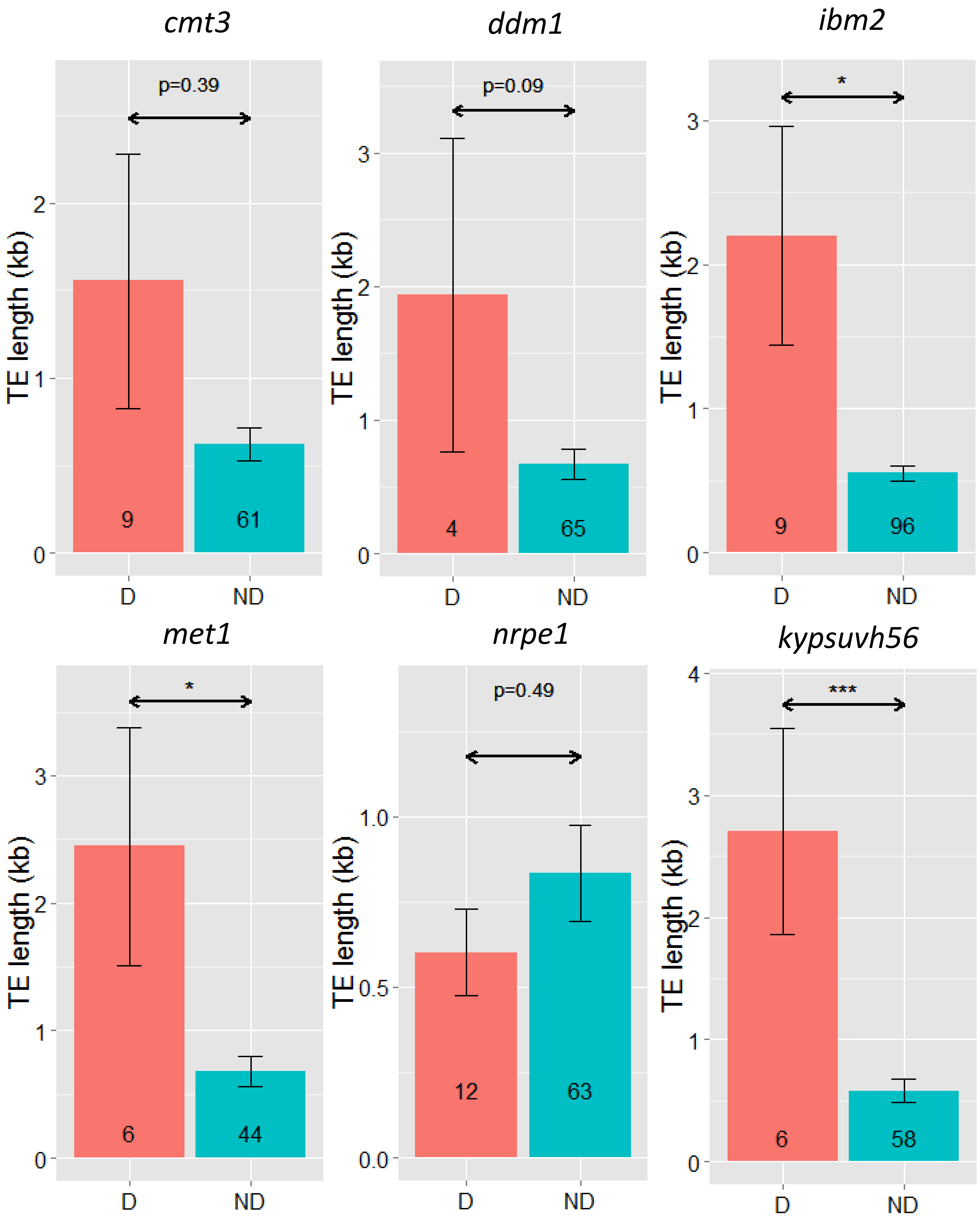

Supplementary Figure S13. Expression change at 5' and 3' of genes with either hyper- or hypo-methylated intronic TEs analyzed by Real-time PCR. Bars represent means of three technical replicates ± s.e.m. Regions in each genes analyzed by Real-time PCR are indicated in Figure 7.

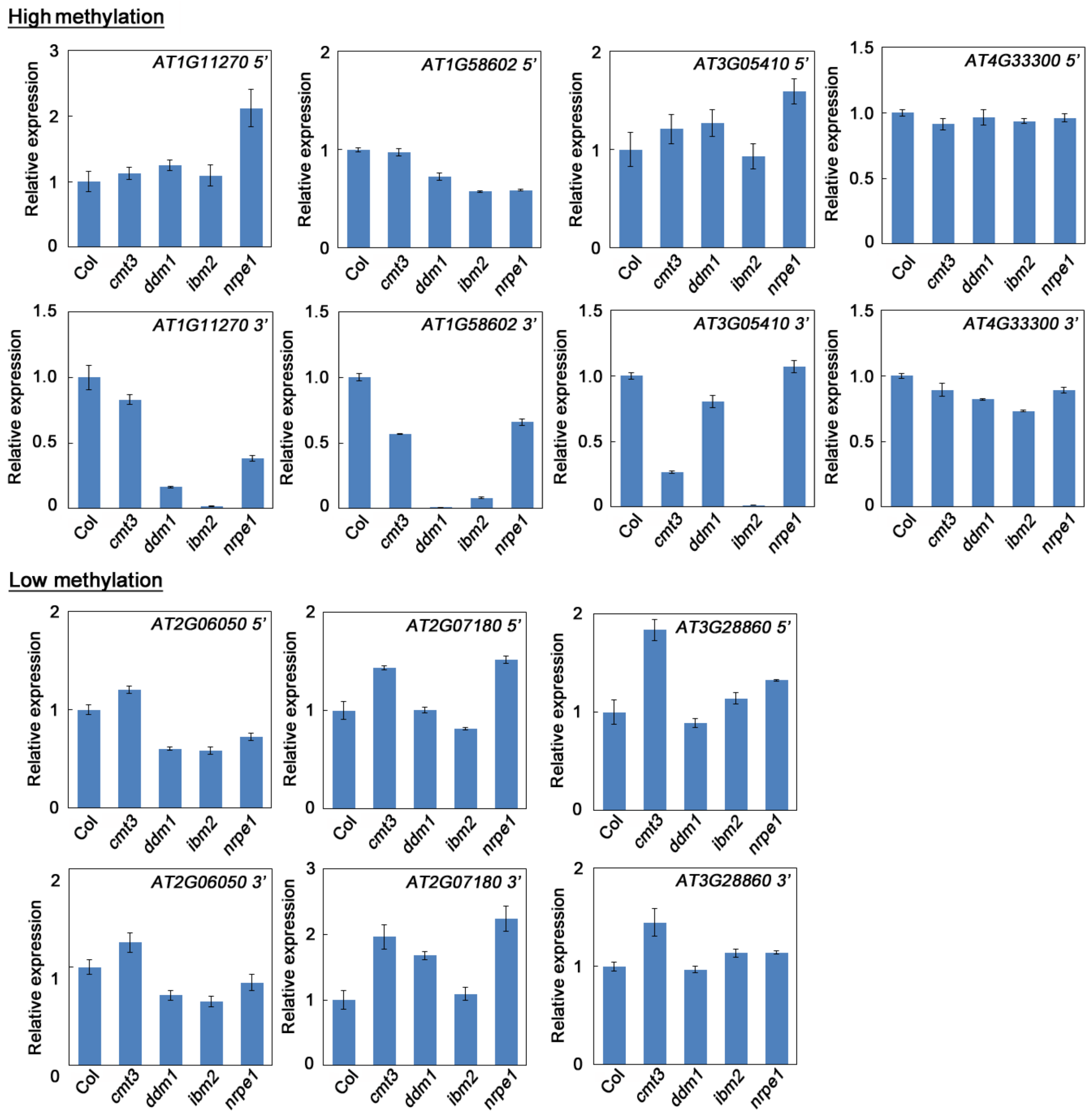

Supplementary Figure S14. Epigenetic factors are required for DNA methylation of intronic TEs. Bisulfite sequencing analysis for DNA methylation in *AT1G11270* (A), *AT3G05410* (B) and *AT4G33300* (*ADR1-L1*) in epigenetic mutants, shown as Figure 7A, B. For each genotype, ten to twelve independent clones were sequenced.

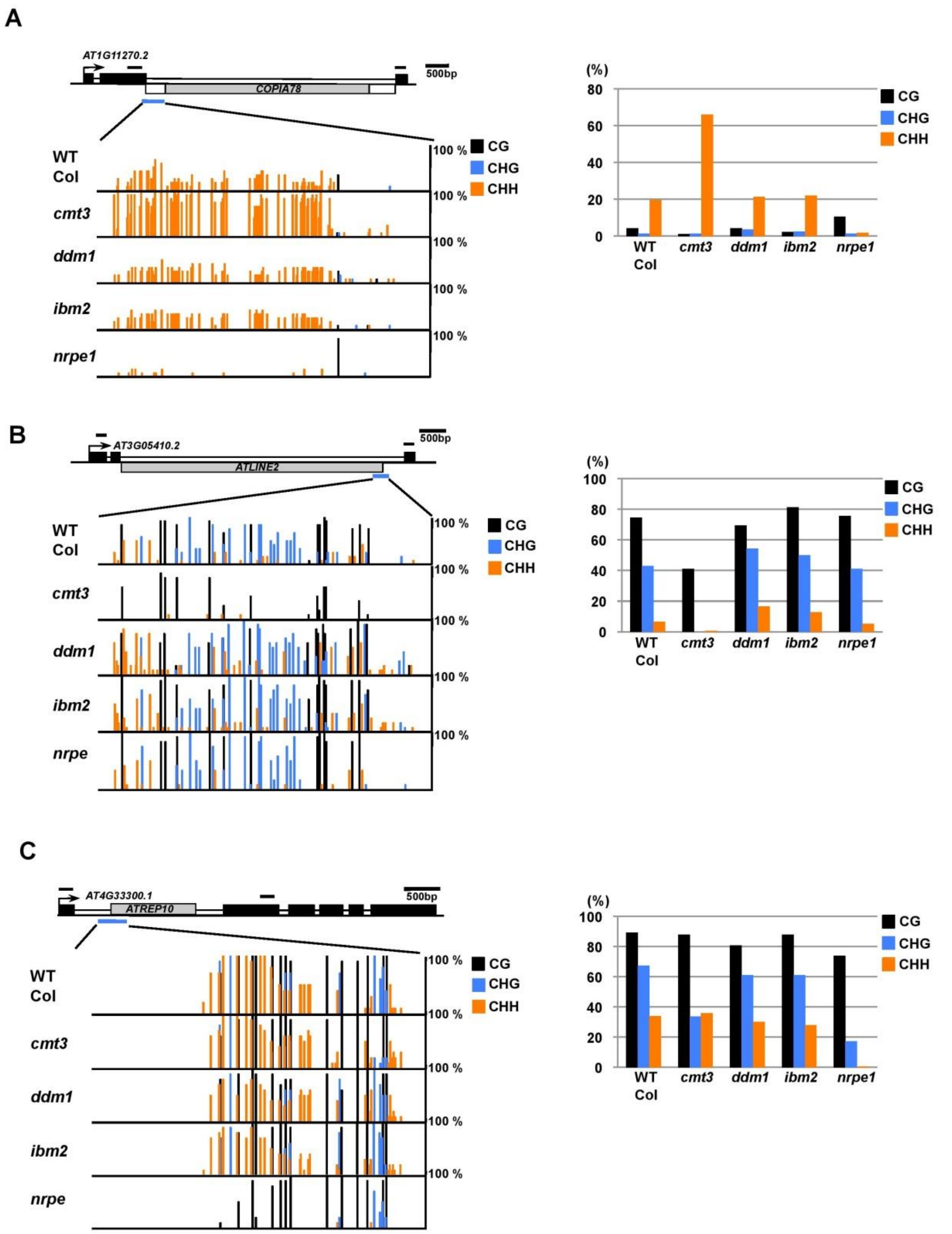

Supplement: SUPPLEMENTARY DATA [file supp_gkv258_nar-00113-x-2015-File016.pdf]
